# Characteristics of predictor sets found using differential prioritization

**DOI:** 10.1186/1748-7188-2-7

**Published:** 2007-06-04

**Authors:** Chia Huey Ooi, Madhu Chetty, Shyh Wei Teng

**Affiliations:** 1 Gippsland School of Information Technology, Monash University, Churchill, VIC 3842, Australia

## Abstract

**Background:**

Feature selection plays an undeniably important role in classification problems involving high dimensional datasets such as microarray datasets. For filter-based feature selection, two well-known criteria used in forming predictor sets are relevance and redundancy. However, there is a third criterion which is at least as important as the other two in affecting the efficacy of the resulting predictor sets. This criterion is the degree of differential prioritization (DDP), which varies the emphases on relevance and redundancy depending on the value of the DDP. Previous empirical works on publicly available microarray datasets have confirmed the effectiveness of the DDP in molecular classification. We now propose to establish the fundamental strengths and merits of the DDP-based feature selection technique. This is to be done through a simulation study which involves vigorous analyses of the characteristics of predictor sets found using different values of the DDP from toy datasets designed to mimic real-life microarray datasets.

**Results:**

A simulation study employing analytical measures such as the distance between classes before and after transformation using principal component analysis is implemented on toy datasets. From these analyses, the necessity of adjusting the differential prioritization based on the dataset of interest is established. This conclusion is supported by comparisons against both simplistic rank-based selection and state-of-the-art equal-priorities scoring methods, which demonstrates the superiority of the DDP-based feature selection technique. Reapplying similar analyses to real-life multiclass microarray datasets provides further confirmation of our findings and of the significance of the DDP for practical applications.

**Conclusion:**

The findings have been achieved based on analytical evaluations, not empirical evaluation involving classifiers, thus providing further basis for the usefulness of the DDP and validating the need for unequal priorities on relevance and redundancy during feature selection for microarray datasets, especially highly multiclass datasets.

## Background

The aim of feature selection is to form, from all available features in a dataset, a relatively small subset of features capable of producing the optimal classification accuracy. This subset is called the predictor set. A feature selection technique is made of two components: the predictor set scoring method (which evaluates the goodness of a candidate predictor set); and the search method (which searches the gene subset space for the predictor set based on the scoring method). The technique becomes wrapper-based when classifiers are invoked in the predictor set scoring method. Otherwise, the technique is filter-based, which is the focus of this study.

An important principle behind most filter-based feature selection studies can be summarized by the following statement: A good predictor set should contain features highly correlated to the target class concept, and yet uncorrelated with each other [1]. The predictor set attribute referred to in the first part of this statement, 'relevance', is the backbone of rank-based feature selection techniques. The aspect alluded to in the second part, 'redundancy', refers to pairwise relationships between all pairs of features in the predictor set. The relevance of a predictor set tells us how well the predictor set is able to distinguish among different classes. The redundancy in a predictor set indicates the amount of similarity among the members of the predictor set, or rather, the amount of repetitions in terms of the information conveyed by the members of the predictor set.

Previous studies [1,2] have based their feature selection techniques on the concept of relevance and redundancy having equal importance in the formation of a good predictor set. We call the predictor set scoring methods used in such correlation-based feature selection techniques *equal-priorities scoring methods*. On the other hand, it is demonstrated in [3] using a 2-class problem that seemingly redundant features may improve the discriminant power of the predictor set instead, although it remains to be seen how this scales up to multiclass domains with thousands of features. A study was implemented on the effect of varying the importance of minimizing redundancy in predictor set evaluation in [4]. However, due to its use of a relevance score that is inapplicable to multiclass problems, the study was limited to only binary classification.

Currently, when it comes to the use of filter-based feature selection for multiclass molecular classification, three popular recommendations are: 1) no selection [5,6]; 2) select based on relevance alone [5,7]; and finally, 3) select based on relevance and redundancy [2,8]. Thus, so far, relevance and redundancy are the two existing criteria which have ever been used in predictor set scoring methods for multiclass molecular classification.

To these two criteria we introduce one modification and a new criterion in our previous study [9]:

• Antiredundancy, which is a parameter opposite to redundancy in terms of quality and thus is to be maximized along with relevance. Accordingly, instead of maximizing relevance and minimizing redundancy, we now maximize both relevance and antiredundancy.

• Aside from relevance and antiredundancy/redundancy, there is a third criterion in feature selection which is necessary for the formation of the predictor set. The third criterion is the degree of differential prioritization (DDP), which represents the relative importance placed between relevance and antiredundancy.

DDP compels the search method to prioritize the optimization of one of the two criteria (of relevance or antiredundancy) at the cost of the optimization of the other. In other words, DDP controls the balance between the two requirements in feature selection (maximizing relevance and maximizing antiredundancy). Therefore, unlike other existing correlation-based techniques, the novelty of the DDP-based feature selection technique is that it does not take for granted that the optimizations of both elements of relevance and antiredundancy are to have equal priorities in the search for the predictor set [10,11].

DDP is represented by a variable *α *which can take any value from 0 to 1. Decreasing the value of *α *forces the search method to put more priority on maximizing antiredundancy at the cost of maximizing relevance. Raising the value of *α *increases the emphasis on maximizing relevance (and at the same time decreases the emphasis on maximizing antiredundancy) during the search for the predictor set [10,11].

A predictor set found using a larger value of *α *contains more features with strong relevance to the target class concept, but also more redundancy among these features. Conversely, a predictor set obtained using a smaller value of *α *contains less redundancy among its member features, but at the same time also has fewer features with strong relevance to the target class concept. At *α *= 0.5, we get an equal-priorities scoring method. At *α *= 1, the feature selection technique becomes rank-based. Thus, the beauty of the DDP concept is that it subsumes the two existing concepts in feature selection which are represented by equal-priorities scoring methods and rank-based techniques.

A large body of our work has provided empirical support regarding the efficacy of the DDP concept in feature selection [9-12], including comparisons to other feature selection techniques on highly multiclass microarray datasets in [11]. However, we have yet to establish the fundamental strengths and merits of the DDP-based feature selection technique. This is precisely the aim of this paper, which is to be realized through a simulation study involving vigorous analyses of predictor sets found using the DDP-based feature selection technique and simple but illustrative examples using toy datasets.

To generate toy datasets for this purpose, we employ two models which are well-known and recognized not only in the domains of molecular classification and microarray analysis but also conventional data mining [12]. Later in this paper, we also show how close conditions in real-life multiclass microarray datasets resemble those of our toy datasets. Additional advantages of toy datasets include the unlimited number of datasets we can generate (vs. the limited number of available real-life microarray datasets [12]); the control we are able to exercise over dataset characteristics such as the number of classes and features; and prior knowledge of the members of the ideal predictor set, which provides the ultimate means for measuring the efficacy of the feature selection technique without involving the inductions of actual classifiers.

The organization of the paper is as follows: Beginning with descriptions of the models used to produce the toy datasets: the OVA (one-vs.-all) and PW (pairwise) models, we proceed to analyze the characteristics of the predictor sets obtained from each of the toy datasets and then summarize the properties of the predictor sets which are dependent on the associated DDP values. After reapplying the same set of analyses to eight real-life multiclass microarray datasets, we demonstrate how the DDP works for datasets with different number of classes. We then follow with further discussion of the results and present the conclusions of the study. Finally, in the Methods section, we describe the DDP-based feature selection technique and the real-life datasets used in this study.

## Results

### Toy datasets

The aim of toy datasets is to provide simple but clear and demonstrative examples on the importance of the correct choice of the value of the DDP in forming the best predictor set. Furthermore, another advantage of toy datasets is the fact that we know exactly just how large a predictor set should be for each case, facilitating the task of determining the value of the maximum size of the predictor set, *P*.

It is widely accepted that over-expression or under-expression (suppression) of genes causes the difference in phenotype among samples of different classes. The categorization of gene expression is given as follows.

• A gene is over-expressed: if its expression value is above baseline.

• A gene is under-expressed: if its expression value is below baseline.

• Baseline interval: the normal range of expression value.

As one of the data processing steps recommended in [13], logarithmic transformation are applied on microarray datasets: base 10 log for data derived from oligonucleotide (Affymetrix) platform and base 2 log for data derived from cDNA (two-color) platform. Later, another of the data processing steps, normalization, is conducted. Normalization involves the standardization of the gene expression data by mean-centering so that the samples have mean 0 across genes [13]. The purpose of normalization is to prevent the expression levels in one particular sample from dominating the average expression levels across samples [14]. (This normalization is not to be confused with dye normalization, which is performed in an earlier stage of data processing.)

Since the result of normalization is that the mean expression across all genes in a sample is 0, the 'average' genes in a sample have expression values of or close to 0. As the 'average' genes are associated with the baseline or the normal range of expression, the value 0 denotes the center of the baseline interval. Over-expression is represented by positive values and under-expression by negative values. With this categorization, we next employ two well-known paradigms leading to the OVA and PW models, which are then used to generate two different sets of toy datasets.

### One-vs.-all (OVA) model

The crux of the OVA concept has gained wide, albeit tacit, acceptance among researchers involved in gene expression analysis. The fact that particular genes are only over-expressed in tissues of a certain type of cancer, and not any other types of cancer or normal tissues [6], is part of the domain knowledge. Hence the term 'marker' – for genes that mark the particular cancer associated with them. In the OVA model, certain groups of genes, also called the 'marker genes' are only over-expressed (or under-expressed) in samples belonging to a particular class and never in samples of other classes. This model emphasizes that a group of marker genes is specific to one class. Therefore for a *K*-class dataset, there are *K *different groups of marker genes.

Let us denote as *G *the number of genes in each group of marker genes, *X*_max _and *X*_min _the maximum and minimum limits, respectively, to the absolute value of the class means for the whole dataset. Thus, for the *g*-th gene in a group of marker genes, the maximum limit to the absolute value of the class means is defined as:

*x*_max,*g *_= *X*_max _- (Δ*X*)(*g *- 1)

where *g *= 1, 2, ..., *G*, and

ΔX=Xmax⁡−Xmin⁡G−1
 MathType@MTEF@5@5@+=feaafiart1ev1aaatCvAUfKttLearuWrP9MDH5MBPbIqV92AaeXatLxBI9gBaebbnrfifHhDYfgasaacH8akY=wiFfYdH8Gipec8Eeeu0xXdbba9frFj0=OqFfea0dXdd9vqai=hGuQ8kuc9pgc9s8qqaq=dirpe0xb9q8qiLsFr0=vr0=vr0dc8meaabaqaciaacaGaaeqabaqabeGadaaakeaacqqHuoarcqWGybawcqGH9aqpdaWcaaqaaiabdIfaynaaBaaaleaacyGGTbqBcqGGHbqycqGG4baEaeqaaOGaeyOeI0IaemiwaG1aaSbaaSqaaiGbc2gaTjabcMgaPjabc6gaUbqabaaakeaacqWGhbWrcqGHsislcqaIXaqmaaaaaa@3F68@

For the *g*-th gene in a group of marker genes, the difference between the class means of subsequent classes is defined in the following manner:

Δxg=2xmax⁡,gK−1
 MathType@MTEF@5@5@+=feaafiart1ev1aaatCvAUfKttLearuWrP9MDH5MBPbIqV92AaeXatLxBI9gBaebbnrfifHhDYfgasaacH8akY=wiFfYdH8Gipec8Eeeu0xXdbba9frFj0=OqFfea0dXdd9vqai=hGuQ8kuc9pgc9s8qqaq=dirpe0xb9q8qiLsFr0=vr0=vr0dc8meaabaqaciaacaGaaeqabaqabeGadaaakeaacqqHuoarcqWG4baEdaWgaaWcbaGaem4zaCgabeaakiabg2da9maalaaabaGaeGOmaiJaemiEaG3aaSbaaSqaaiGbc2gaTjabcggaHjabcIha4jabcYcaSiabdEgaNbqabaaakeaacqWGlbWscqGHsislcqaIXaqmaaaaaa@3E28@

The purpose of equations (1), (2), and (3) is to produce the following effect: We would like to vary the class means such that there is an imbalance or inequality in terms of class means among the *K *classes. The reasons are firstly to mimic a condition prevalent in multiclass microarray datasets (imbalance among classes in terms of class means even after normalization), especially in datasets with large number of classes; and secondly, to present a challenge to the feature selection technique in choosing sufficiently relevant but non-redundant genes. We will provide further elucidation on the second reason later in this section.

Another purpose of the equations is to generate genes with varying relevance in each group of marker genes. Based on equations (1), (2), and (3), the first gene in a group of marker genes (the gene associated with *g *= 1) has the strongest relevance among the members of that group of marker genes. Accordingly, the gene with the weakest relevance is the last gene in a group of marker genes (the gene associated with *g *= *G*). The reason for doing this is also to present a challenge to the feature selection technique in choosing sufficiently relevant but non-redundant genes.

Next, initialize a matrix *M*: = (*μ*_*i*, *k*_)_*N *× *K *_of zeros where *N *is the total number of genes in the dataset, and, in this case, is the product of *G *and *K*. This is the matrix of class means, whose element, *μ*_*i*,*k*_, represents the mean of gene *i *across samples belonging to class *k *(*k *= 1, 2, ..., *K*):

*μ*_(*g *- 1)*K *+ *k*, *k *_= (-1^*g*^)[*x*_max, *g *_- (Δ*x*_*g*_)(*k *- 1)]

The [(*g *- 1)*K *+ *k*]-th gene is the *g*-th member of the *k*-th group of marker genes and therefore has non-zero class mean for class *k *and zero class means for all other classes – the archetypal OVA trait. The term (-1^*g*^) serves to change the sign of the class mean at different values of *g *so as to produce both over- and under-expressed marker genes.

Standard deviation among samples of the same class, or class standard deviation, is set to 1 for all instances, *σ*_*i*,*k *_= 1 for all *k *and *i*. For all *k*, a total of *m *samples are generated for class *k *using Gaussian distribution of mean *μ*_*i*,*k *_and standard deviation *σ*_*i*,*k *_for gene *i*.

In Table 1, an entry on the *i*-th row and *k*-th column represents the class mean of class *k *for gene *i*, where *i *= [(*g *- 1)*K *+ *k*], and therefore gene *i *is the *g*-th member of the *k*-th group of marker genes. We can see that using relevance alone as a criterion, and with uniform class size, marker genes associated with class 1 and 4 will always be favored more than marker genes specific to any other classes, regardless of the value of *g*. Including antiredundancy as the second criterion will obviate this imbalanced predilection – therein lies the reason for us to use unequal values for class means among different classes. But how much weight is to be assigned to relevance, and how much to antiredundancy?

**Table 1:** A 4-class example from the OVA model. *μ*_*i*, *k *_represents the mean of gene *i *across samples belonging to class *k*.

*g*	*k*	*μ*_*i*,*k*_	*μ*_*i*,1_	*μ*_*i*,2_	*μ*_*i*,3_	*μ*_*i*,4_
1	1	*μ*_1,*k*_	-*X*_max_	0	0	0
1	2	*μ*_2,*k*_	0	-0.5 *X*_max_	0	0
1	3	*μ*_3,*k*_	0	0	0.5 *X*_max_	0
1	4	*μ*_4,*k*_	0	0	0	*X*_max_
2	1	*μ*_5,*k*_	*X*_max_-Δ*X*	0	0	0
2	2	*μ*_6,*k*_	0	0.5(*X*_max_-Δ*X*)	0	0
2	3	*μ*_7,*k*_	0	0	0.5(Δ*X *- *X*_max_)	0
2	4	*μ*_8,*k*_	0	0	0	Δ*X *- *X*_max_
⋮	⋮	⋮	⋮	⋮	⋮	⋮
*G*	1	*μ*_(*G *- 1)*K*+1,*k*_	(-1^*G*^)*X*_min_	0	0	0
*G*	2	*μ*_(*G *- 1)*K*+2,*k*_	0	0.5(-1^*G*^) *X*_min_	0	0
*G*	3	*μ*_(*G *- 1)*K*+3,*k*_	0	0	-0.5(-1^*G*^) *X*_min_	0
*G*	4	*μ*_(*G *- 1)*K*+4,*k*_	0	0	0	-(-1^*G*^) *X*_min_

The ostensible answer would be equal weights, which is the foundation of existing equal-priorities scoring methods. But as mentioned previously in the Background section, it has been implied that antiredundancy is not as important as relevance for the 2-class problem [3] – this is obvious in case of our OVA toy dataset; any subset of sufficiently relevant genes is capable of differentiating between the two classes. Hence we ask the questions which motivate the concept of the DDP: If at *K *= 2, antiredundancy is not as important as relevance, will this change as the number of classes increases (an important theme in multiclass classification studies)? As *K *increases, might not the importance of antiredundancy (w.r.t. relevance) increase as well? If yes, is there a point where antiredundancy eventually overcomes relevance in terms of importance as a criterion in feature selection? These questions are to be answered from the analyses in this study.

### Pairwise (PW) model

In the PW model, for a given pair of classes, a group of marker genes only distinguishes samples from one class of the pair of classes against samples from the other class of the pair of classes. As implied by its name, this model represents the 1-vs.-1 paradigm as opposed to the 1-vs.-others of the OVA model. For a *K*-class dataset, there are (K2)
 MathType@MTEF@5@5@+=feaafiart1ev1aaatCvAUfKttLearuWrP9MDH5MBPbIqV92AaeXatLxBI9gBaebbnrfifHhDYfgasaacH8akY=wiFfYdH8Gipec8Eeeu0xXdbba9frFj0=OqFfea0dXdd9vqai=hGuQ8kuc9pgc9s8qqaq=dirpe0xb9q8qiLsFr0=vr0=vr0dc8meaabaqaciaacaGaaeqabaqabeGadaaakeaadaqadaqaauaabeqaceaaaeaacqWGlbWsaeaacqaIYaGmaaaacaGLOaGaayzkaaaaaa@3053@ different groups of marker genes in the PW model. (K2)=K⋅(K−1)2
 MathType@MTEF@5@5@+=feaafiart1ev1aaatCvAUfKttLearuWrP9MDH5MBPbIqV92AaeXatLxBI9gBaebbnrfifHhDYfgasaacH8akY=wiFfYdH8Gipec8Eeeu0xXdbba9frFj0=OqFfea0dXdd9vqai=hGuQ8kuc9pgc9s8qqaq=dirpe0xb9q8qiLsFr0=vr0=vr0dc8meaabaqaciaacaGaaeqabaqabeGadaaakeaadaqadaqaauaabeqaceaaaeaacqWGlbWsaeaacqaIYaGmaaaacaGLOaGaayzkaaGaeyypa0ZaaSaaaeaacqWGlbWscqGHflY1cqGGOaakcqWGlbWscqGHsislcqaIXaqmcqGGPaqkaeaacqaIYaGmaaaaaa@3A72@ is the number of unique pairs of classes in a *K*-class dataset; it is also known as ^*K*^*C*_2_.

As is the case in the OVA model, we denote as *G *the number of genes in each group of marker genes, *X*_max _and *X*_min _the maximum and minimum limits, respectively, to the absolute value of the class means for the whole dataset. The definitions of *x*_max,*g*_, Δ*X*, and Δ*x*_*g *_are the same as for the OVA model.

Initialize a matrix *M*: = (*μ*_*i*,*k*_)_*N *× *K *_of zeros where *N *is the total number of genes in the dataset, and, in this case, is the product of *G *and (K2)
 MathType@MTEF@5@5@+=feaafiart1ev1aaatCvAUfKttLearuWrP9MDH5MBPbIqV92AaeXatLxBI9gBaebbnrfifHhDYfgasaacH8akY=wiFfYdH8Gipec8Eeeu0xXdbba9frFj0=OqFfea0dXdd9vqai=hGuQ8kuc9pgc9s8qqaq=dirpe0xb9q8qiLsFr0=vr0=vr0dc8meaabaqaciaacaGaaeqabaqabeGadaaakeaadaqadaqaauaabeqaceaaaeaacqWGlbWsaeaacqaIYaGmaaaacaGLOaGaayzkaaaaaa@3053@. Again this is the matrix of class means, whose element, *μ*_*i*,*k*_, represents the mean of gene *i *across samples belonging to class *k*. Now let us define the *q*-th pair of classes as *C*_*q *_= {*c*_1,*q*_,*c*_2,*q*_} where *q *= 1, 2,..., (K2)
 MathType@MTEF@5@5@+=feaafiart1ev1aaatCvAUfKttLearuWrP9MDH5MBPbIqV92AaeXatLxBI9gBaebbnrfifHhDYfgasaacH8akY=wiFfYdH8Gipec8Eeeu0xXdbba9frFj0=OqFfea0dXdd9vqai=hGuQ8kuc9pgc9s8qqaq=dirpe0xb9q8qiLsFr0=vr0=vr0dc8meaabaqaciaacaGaaeqabaqabeGadaaakeaadaqadaqaauaabeqaceaaaeaacqWGlbWsaeaacqaIYaGmaaaacaGLOaGaayzkaaaaaa@3053@, *c*_1,*q *_∈ [1, *K*], *c*_2,*q *_∈ [1, *K*], and *c*_1,*q *_≠ *c*_2,*q*_. For the *q*-th pair of classes, the class means are computed as follows:

μ(g−1)⋅(K2)+q,cb,q=(−1g)[xmax⁡,g−(Δxg)(cb,q−1)]
 MathType@MTEF@5@5@+=feaafiart1ev1aaatCvAUfKttLearuWrP9MDH5MBPbIqV92AaeXatLxBI9gBaebbnrfifHhDYfgasaacH8akY=wiFfYdH8Gipec8Eeeu0xXdbba9frFj0=OqFfea0dXdd9vqai=hGuQ8kuc9pgc9s8qqaq=dirpe0xb9q8qiLsFr0=vr0=vr0dc8meaabaqaciaacaGaaeqabaqabeGadaaakeaaiiGacqWF8oqBdaWgaaWcbaGaeiikaGIaem4zaCMaeyOeI0IaeGymaeJaeiykaKIaeyyXIC9aaeWaaeaafaqabeGabaaabaGaem4saSeabaGaeGOmaidaaaGaayjkaiaawMcaaiabgUcaRiabdghaXjabcYcaSiabdogaJnaaBaaameaacqWGIbGycqGGSaalcqWGXbqCaeqaaaWcbeaakiabg2da9maabmaabaGaeyOeI0IaeGymaeZaaWbaaSqabeaacqWGNbWzaaaakiaawIcacaGLPaaadaWadaqaaiabdIha4naaBaaaleaacyGGTbqBcqGGHbqycqGG4baEcqGGSaalcqWGNbWzaeqaaOGaeyOeI0YaaeWaaeaacqqHuoarcqWG4baEdaWgaaWcbaGaem4zaCgabeaaaOGaayjkaiaawMcaamaabmaabaGaem4yam2aaSbaaSqaaiabdkgaIjabcYcaSiabdghaXbqabaGccqGHsislcqaIXaqmaiaawIcacaGLPaaaaiaawUfacaGLDbaaaaa@611F@

for *b *= 1 and *b *= 2. For the PW model, the [(g−1)⋅(K2)+q]
 MathType@MTEF@5@5@+=feaafiart1ev1aaatCvAUfKttLearuWrP9MDH5MBPbIqV92AaeXatLxBI9gBaebbnrfifHhDYfgasaacH8akY=wiFfYdH8Gipec8Eeeu0xXdbba9frFj0=OqFfea0dXdd9vqai=hGuQ8kuc9pgc9s8qqaq=dirpe0xb9q8qiLsFr0=vr0=vr0dc8meaabaqaciaacaGaaeqabaqabeGadaaakeaadaWadaqaamaabmaabaGaem4zaCMaeyOeI0IaeGymaedacaGLOaGaayzkaaGaeyyXIC9aaeWaaeaafaqabeGabaaabaGaem4saSeabaGaeGOmaidaaaGaayjkaiaawMcaaiabgUcaRiabdghaXbGaay5waiaaw2faaaaa@3B99@-th gene is the *g*-th member of the *q*-th group of marker genes and therefore has non-zero class means for classes *c*_1,*q *_and *c*_2,*q*_, and zero class means for all other classes – which is the typical PW characteristic.

The procedure for the generation of datasets is similar to that of the OVA model.

### Experiment settings

In this study, for both models, *X*_max _and *X*_min _are set to 100 and 1 respectively, while the number of samples per class, or class size, *m*, is set to 100 uniformly for all classes.

Ten values of *α *are tested from 0.1 to 1 with equal intervals of 0.1, *α *denoting the value of the DDP. For both models, the number of genes in each group of marker genes, *G*, is set to 3, 5, 10, 20, and 30. We test for *K *= 2 to *K *= 30, *K *denoting the number of classes in a dataset. Since no inductions of classifiers are to be implemented in this study, whole datasets are used as training sets during feature selection.

For toy datasets generated from the OVA model, the minimum predictor set size necessary to differentiate among the *K *classes is *K *- 1. The optimal predictor set is actually any subset of *K *- 1 genes from the first *K *of the marker genes (i.e., at *g *= 1) generated using the class means defined in equation (4).

In case of toy datasets based on the PW model, the optimal predictor set is any subset *S *of *K *- 2 genes from the first (K2)
 MathType@MTEF@5@5@+=feaafiart1ev1aaatCvAUfKttLearuWrP9MDH5MBPbIqV92AaeXatLxBI9gBaebbnrfifHhDYfgasaacH8akY=wiFfYdH8Gipec8Eeeu0xXdbba9frFj0=OqFfea0dXdd9vqai=hGuQ8kuc9pgc9s8qqaq=dirpe0xb9q8qiLsFr0=vr0=vr0dc8meaabaqaciaacaGaaeqabaqabeGadaaakeaadaqadaqaauaabeqaceaaaeaacqWGlbWsaeaacqaIYaGmaaaacaGLOaGaayzkaaaaaa@3053@ of the marker genes generated using the class means defined in equation (5) at *g *= 1 which also fulfills the following condition:

(∪i∈SCqi)∩{1,2,...,K}={1,2,...,K}
 MathType@MTEF@5@5@+=feaafiart1ev1aaatCvAUfKttLearuWrP9MDH5MBPbIqV92AaeXatLxBI9gBaebbnrfifHhDYfgasaacH8akY=wiFfYdH8Gipec8Eeeu0xXdbba9frFj0=OqFfea0dXdd9vqai=hGuQ8kuc9pgc9s8qqaq=dirpe0xb9q8qiLsFr0=vr0=vr0dc8meaabaqaciaacaGaaeqabaqabeGadaaakeaadaqadaqaamaatafabaGaem4qam0aaSbaaSqaaiabdghaXnaaBaaameaacqWGPbqAaeqaaaWcbeaaaeaacqWGPbqAcqGHiiIZcqWGtbWuaeqaniablQIivbaakiaawIcacaGLPaaacqGHPiYXdaGadeqaaiabigdaXiabcYcaSiabikdaYiabcYcaSiabc6caUiabc6caUiabc6caUiabcYcaSiabdUealbGaay5Eaiaaw2haaiabg2da9maacmqabaGaeGymaeJaeiilaWIaeGOmaiJaeiilaWIaeiOla4IaeiOla4IaeiOla4IaeiilaWIaem4saSeacaGL7bGaayzFaaaaaa@4FD1@

where |*S*| = *K *- 2, qi=remainder[i(K2)]
 MathType@MTEF@5@5@+=feaafiart1ev1aaatCvAUfKttLearuWrP9MDH5MBPbIqV92AaeXatLxBI9gBaebbnrfifHhDYfgasaacH8akY=wiFfYdH8Gipec8Eeeu0xXdbba9frFj0=OqFfea0dXdd9vqai=hGuQ8kuc9pgc9s8qqaq=dirpe0xb9q8qiLsFr0=vr0=vr0dc8meaabaqaciaacaGaaeqabaqabeGadaaakeaacqWGXbqCdaWgaaWcbaGaemyAaKgabeaakiabg2da9iabdkhaYjabdwgaLjabd2gaTjabdggaHjabdMgaPjabd6gaUjabdsgaKjabdwgaLjabdkhaYnaadmaabaWaaSaaaeaacqWGPbqAaeaadaqadaqaauaabeqaceaaaeaacqWGlbWsaeaacqaIYaGmaaaacaGLOaGaayzkaaaaaaGaay5waiaaw2faaaaa@43F1@, i=[(g−1)⋅(K2)+q]
 MathType@MTEF@5@5@+=feaafiart1ev1aaatCvAUfKttLearuWrP9MDH5MBPbIqV92AaeXatLxBI9gBaebbnrfifHhDYfgasaacH8akY=wiFfYdH8Gipec8Eeeu0xXdbba9frFj0=OqFfea0dXdd9vqai=hGuQ8kuc9pgc9s8qqaq=dirpe0xb9q8qiLsFr0=vr0=vr0dc8meaabaqaciaacaGaaeqabaqabeGadaaakeaacqWGPbqAcqGH9aqpdaWadaqaamaabmaabaGaem4zaCMaeyOeI0IaeGymaedacaGLOaGaayzkaaGaeyyXIC9aaeWaaeaafaqabeGabaaabaGaem4saSeabaGaeGOmaidaaaGaayjkaiaawMcaaiabgUcaRiabdghaXbGaay5waiaaw2faaaaa@3DFA@, and *C*_*qi *_represents the *q*_*i*_-th pair of classes as defined previously in the subsection on the PW model. In other words, the optimal predictor set contains representatives from enough groups of marker genes such that all *K *classes are represented in pairs of classes associated to those groups of marker genes.

Therefore for datasets generated from the OVA model, *P *is set to *K *- 1 and for those from the PW model, *P *is set to *K *- 2.

### Separation of classes

A natural way to measure separation of classes is the distance between pairs of class centers. We use two popular metrics, the Euclidean and the Manhattan (or taxicab) distances. At the end of the "One-vs.-all (OVA) model" subsection under the Results section, we discuss a preceding study on feature selection [3] which inspired the DDP concept. The authors of that study employ a form of separation of classes to demonstrate that a redundant feature may enhance the predictor set's ability to distinguish between two classes in a 2-class problem (thus implying that antiredundancy is not as important as relevance for the 2-class problem). This form of separation of classes corresponds to the Manhattan distance used in our study.

In a 2-class problem, the authors of [3] first present two features from a toy dataset which are both relevant but redundant w.r.t each other, contained in a predictor set distinguishing between the two classes. Then, after a 45° rotation of those two features, the authors of that study show that the Manhattan distance between the class centers along one axis is now greater by a factor of 2
 MathType@MTEF@5@5@+=feaafiart1ev1aaatCvAUfKttLearuWrP9MDH5MBPbIqV92AaeXatLxBI9gBaebbnrfifHhDYfgasaacH8akY=wiFfYdH8Gipec8Eeeu0xXdbba9frFj0=OqFfea0dXdd9vqai=hGuQ8kuc9pgc9s8qqaq=dirpe0xb9q8qiLsFr0=vr0=vr0dc8meaabaqaciaacaGaaeqabaqabeGadaaakeaadaGcaaqaaiabikdaYaWcbeaaaaa@2DB9@ than the corresponding Manhattan distance in the original plane – thus increasing the separation of classes. For a predictor set with two members, the aforementioned 45° rotation is akin to the transformation by principal component analysis, which we will implement later in this study.

We observe that the Euclidean distance remains the same before and after the transformation in that study. Therefore we have included the Euclidean distance as another form of separation of classes to study, if any, the differences between the two distances in the context of the DDP. Moreover, the Euclidean distance is as popularly used as the Manhattan distance in the field of intelligent data analysis.

For the *q*-th pair of classes, *C*_*q *_= {*c*_1,*q*_, *c*_2,*q*_} (where in a *K*-class problem *q *= 1, 2,..., (K2)
 MathType@MTEF@5@5@+=feaafiart1ev1aaatCvAUfKttLearuWrP9MDH5MBPbIqV92AaeXatLxBI9gBaebbnrfifHhDYfgasaacH8akY=wiFfYdH8Gipec8Eeeu0xXdbba9frFj0=OqFfea0dXdd9vqai=hGuQ8kuc9pgc9s8qqaq=dirpe0xb9q8qiLsFr0=vr0=vr0dc8meaabaqaciaacaGaaeqabaqabeGadaaakeaadaqadaqaauaabeqaceaaaeaacqWGlbWsaeaacqaIYaGmaaaacaGLOaGaayzkaaaaaa@3053@, *c*_1,*q *_∈ [1, *K*], *c*_2,*q *_∈ [1, *K*], and *c*_1,*q *_≠ *c*_2,*q*_), the separation between classes given by the predictor set found through a DDP value of *α*, *S*_*α*_, measured using the Euclidean metric is given below:

dE,α(c1,q,c2,q)=∑i∈Sα|x¯i,c1,q−x¯i,c2,q|2
 MathType@MTEF@5@5@+=feaafiart1ev1aaatCvAUfKttLearuWrP9MDH5MBPbIqV92AaeXatLxBI9gBaebbnrfifHhDYfgasaacH8akY=wiFfYdH8Gipec8Eeeu0xXdbba9frFj0=OqFfea0dXdd9vqai=hGuQ8kuc9pgc9s8qqaq=dirpe0xb9q8qiLsFr0=vr0=vr0dc8meaabaqaciaacaGaaeqabaqabeGadaaakeaacqWGKbazdaWgaaWcbaGaemyrauKaeiilaWccciGae8xSdegabeaakmaabmaabaGaem4yam2aaSbaaSqaaiabigdaXiabcYcaSiabdghaXbqabaGccqGGSaalcqWGJbWydaWgaaWcbaGaeGOmaiJaeiilaWIaemyCaehabeaaaOGaayjkaiaawMcaaiabg2da9maakaaabaWaaabuaeaadaabdaqaaiqbdIha4zaaraWaaSbaaSqaaiabdMgaPjabcYcaSiabdogaJnaaBaaameaacqaIXaqmcqGGSaalcqWGXbqCaeqaaaWcbeaakiabgkHiTiqbdIha4zaaraWaaSbaaSqaaiabdMgaPjabcYcaSiabdogaJnaaBaaameaacqaIYaGmcqGGSaalcqWGXbqCaeqaaaWcbeaaaOGaay5bSlaawIa7aaWcbaGaemyAaKMaeyicI4Saem4uam1aaSbaaWqaaiab=f7aHbqabaaaleqaniabggHiLdGcdaahaaWcbeqaaiabikdaYaaaaeqaaaaa@5D92@

x¯i,k
 MathType@MTEF@5@5@+=feaafiart1ev1aaatCvAUfKttLearuWrP9MDH5MBPbIqV92AaeXatLxBI9gBaebbnrfifHhDYfgasaacH8akY=wiFfYdH8Gipec8Eeeu0xXdbba9frFj0=OqFfea0dXdd9vqai=hGuQ8kuc9pgc9s8qqaq=dirpe0xb9q8qiLsFr0=vr0=vr0dc8meaabaqaciaacaGaaeqabaqabeGadaaakeaacuWG4baEgaqeamaaBaaaleaacqWGPbqAcqGGSaalcqWGRbWAaeqaaaaa@3203@ is the average of the expression of gene *i *across samples belonging to class *k*. Averaging across all (K2)
 MathType@MTEF@5@5@+=feaafiart1ev1aaatCvAUfKttLearuWrP9MDH5MBPbIqV92AaeXatLxBI9gBaebbnrfifHhDYfgasaacH8akY=wiFfYdH8Gipec8Eeeu0xXdbba9frFj0=OqFfea0dXdd9vqai=hGuQ8kuc9pgc9s8qqaq=dirpe0xb9q8qiLsFr0=vr0=vr0dc8meaabaqaciaacaGaaeqabaqabeGadaaakeaadaqadaqaauaabeqaceaaaeaacqWGlbWsaeaacqaIYaGmaaaacaGLOaGaayzkaaaaaa@3053@ pairs of classes, we obtain the mean Euclidean distance between a pair of classes as measured by *S*_*α*_:

d¯E,α=1θ∑q=1θdE,α(c1,q,c2,q)
 MathType@MTEF@5@5@+=feaafiart1ev1aaatCvAUfKttLearuWrP9MDH5MBPbIqV92AaeXatLxBI9gBaebbnrfifHhDYfgasaacH8akY=wiFfYdH8Gipec8Eeeu0xXdbba9frFj0=OqFfea0dXdd9vqai=hGuQ8kuc9pgc9s8qqaq=dirpe0xb9q8qiLsFr0=vr0=vr0dc8meaabaqaciaacaGaaeqabaqabeGadaaakeaacuWGKbazgaqeamaaBaaaleaacqWGfbqrcqGGSaaliiGacqWFXoqyaeqaaOGaeyypa0ZaaSaaaeaacqaIXaqmaeaacqWF4oqCaaWaaabCaeaacqWGKbazdaWgaaWcbaGaemyrauKaeiilaWIae8xSdegabeaakmaabmaabaGaem4yam2aaSbaaSqaaiabigdaXiabcYcaSiabdghaXbqabaGccqGGSaalcqWGJbWydaWgaaWcbaGaeGOmaiJaeiilaWIaemyCaehabeaaaOGaayjkaiaawMcaaaWcbaGaemyCaeNaeyypa0JaeGymaedabaGae8hUdehaniabggHiLdaaaa@4DEE@

where *θ *denotes (K2)
 MathType@MTEF@5@5@+=feaafiart1ev1aaatCvAUfKttLearuWrP9MDH5MBPbIqV92AaeXatLxBI9gBaebbnrfifHhDYfgasaacH8akY=wiFfYdH8Gipec8Eeeu0xXdbba9frFj0=OqFfea0dXdd9vqai=hGuQ8kuc9pgc9s8qqaq=dirpe0xb9q8qiLsFr0=vr0=vr0dc8meaabaqaciaacaGaaeqabaqabeGadaaakeaadaqadaqaauaabeqaceaaaeaacqWGlbWsaeaacqaIYaGmaaaacaGLOaGaayzkaaaaaa@3053@. Hence, the value of the DDP leading to the best separation of classes in terms of the Euclidean metric is the one which gives the largest d¯E,α
 MathType@MTEF@5@5@+=feaafiart1ev1aaatCvAUfKttLearuWrP9MDH5MBPbIqV92AaeXatLxBI9gBaebbnrfifHhDYfgasaacH8akY=wiFfYdH8Gipec8Eeeu0xXdbba9frFj0=OqFfea0dXdd9vqai=hGuQ8kuc9pgc9s8qqaq=dirpe0xb9q8qiLsFr0=vr0=vr0dc8meaabaqaciaacaGaaeqabaqabeGadaaakeaacuWGKbazgaqeamaaBaaaleaacqWGfbqrcqGGSaaliiGacqWFXoqyaeqaaaaa@31DA@:

αE∗=arg⁡max⁡α(d¯E,α)
 MathType@MTEF@5@5@+=feaafiart1ev1aaatCvAUfKttLearuWrP9MDH5MBPbIqV92AaeXatLxBI9gBaebbnrfifHhDYfgasaacH8akY=wiFfYdH8Gipec8Eeeu0xXdbba9frFj0=OqFfea0dXdd9vqai=hGuQ8kuc9pgc9s8qqaq=dirpe0xb9q8qiLsFr0=vr0=vr0dc8meaabaqaciaacaGaaeqabaqabeGadaaakeaaiiGacqWFXoqydaqhaaWcbaGaemyraueabaGaey4fIOcaaOGaeyypa0ZaaCbeaeaacyGGHbqycqGGYbGCcqGGNbWzcyGGTbqBcqGGHbqycqGG4baEaSqaaiab=f7aHbqabaGcdaqadaqaaiqbdsgaKzaaraWaaSbaaSqaaiabdweafjabcYcaSiab=f7aHbqabaaakiaawIcacaGLPaaaaaa@4257@

The Manhattan distance between the *q*-th pair of classes as measured by *S*_*α *_is computed as follows:

dM,α(c1,q,c2,q)=∑i∈Sα|x¯i,c1,q−x¯i,c2,q|
 MathType@MTEF@5@5@+=feaafiart1ev1aaatCvAUfKttLearuWrP9MDH5MBPbIqV92AaeXatLxBI9gBaebbnrfifHhDYfgasaacH8akY=wiFfYdH8Gipec8Eeeu0xXdbba9frFj0=OqFfea0dXdd9vqai=hGuQ8kuc9pgc9s8qqaq=dirpe0xb9q8qiLsFr0=vr0=vr0dc8meaabaqaciaacaGaaeqabaqabeGadaaakeaacqWGKbazdaWgaaWcbaGaemyta0KaeiilaWccciGae8xSdegabeaakmaabmaabaGaem4yam2aaSbaaSqaaiabigdaXiabcYcaSiabdghaXbqabaGccqGGSaalcqWGJbWydaWgaaWcbaGaeGOmaiJaeiilaWIaemyCaehabeaaaOGaayjkaiaawMcaaiabg2da9maaqafabaWaaqWaaeaacuWG4baEgaqeamaaBaaaleaacqWGPbqAcqGGSaalcqWGJbWydaWgaaadbaGaeGymaeJaeiilaWIaemyCaehabeaaaSqabaGccqGHsislcuWG4baEgaqeamaaBaaaleaacqWGPbqAcqGGSaalcqWGJbWydaWgaaadbaGaeGOmaiJaeiilaWIaemyCaehabeaaaSqabaaakiaawEa7caGLiWoaaSqaaiabdMgaPjabgIGiolabdofatnaaBaaameaacqWFXoqyaeqaaaWcbeqdcqGHris5aaaa@5C69@

Averaging across all (K2)
 MathType@MTEF@5@5@+=feaafiart1ev1aaatCvAUfKttLearuWrP9MDH5MBPbIqV92AaeXatLxBI9gBaebbnrfifHhDYfgasaacH8akY=wiFfYdH8Gipec8Eeeu0xXdbba9frFj0=OqFfea0dXdd9vqai=hGuQ8kuc9pgc9s8qqaq=dirpe0xb9q8qiLsFr0=vr0=vr0dc8meaabaqaciaacaGaaeqabaqabeGadaaakeaadaqadaqaauaabeqaceaaaeaacqWGlbWsaeaacqaIYaGmaaaacaGLOaGaayzkaaaaaa@3053@ pairs of classes, the mean Manhattan distance between a pair of classes is given below:

d¯M,α=1θ∑q=1θdM,α(c1,q−c2,q)
 MathType@MTEF@5@5@+=feaafiart1ev1aaatCvAUfKttLearuWrP9MDH5MBPbIqV92AaeXatLxBI9gBamXvP5wqSXMqHnxAJn0BKvguHDwzZbqegyvzYrwyUfgarqqtubsr4rNCHbGeaGqiA8vkIkVAFgIELiFeLkFeLk=iY=Hhbbf9v8qqaqFr0xc9pk0xbba9q8WqFfeaY=biLkVcLq=JHqVepeea0=as0db9vqpepesP0xe9Fve9Fve9GapdbaqaaeGacaGaaiaabeqaamqadiabaaGcbaGafmizaqMbaebadaWgaaWcbaGaemyta0KaeiilaWccciGae8xSdegabeaakiabg2da9maalaaabaGaeGymaedabaGae8hUdehaamaaqahabaGaemizaq2aaSbaaSqaaiabd2eanjabcYcaSiabeg7aHbqabaaabaGaemyCaeNaeyypa0JaeGymaedabaGae8hUdehaniabggHiLdGcdaqadaqaaiabdogaJnaaBaaaleaacqaIXaqmcqGGSaalcqWGXbqCaeqaaOGaeyOeI0Iaem4yam2aaSbaaSqaaiabikdaYiabcYcaSiabdghaXbqabaaakiaawIcacaGLPaaaaaa@5E54@

where *θ *denotes (K2)
 MathType@MTEF@5@5@+=feaafiart1ev1aaatCvAUfKttLearuWrP9MDH5MBPbIqV92AaeXatLxBI9gBaebbnrfifHhDYfgasaacH8akY=wiFfYdH8Gipec8Eeeu0xXdbba9frFj0=OqFfea0dXdd9vqai=hGuQ8kuc9pgc9s8qqaq=dirpe0xb9q8qiLsFr0=vr0=vr0dc8meaabaqaciaacaGaaeqabaqabeGadaaakeaadaqadaqaauaabeqaceaaaeaacqWGlbWsaeaacqaIYaGmaaaacaGLOaGaayzkaaaaaa@3053@. The value of the DDP which produces the largest d¯M,α
 MathType@MTEF@5@5@+=feaafiart1ev1aaatCvAUfKttLearuWrP9MDH5MBPbIqV92AaeXatLxBI9gBaebbnrfifHhDYfgasaacH8akY=wiFfYdH8Gipec8Eeeu0xXdbba9frFj0=OqFfea0dXdd9vqai=hGuQ8kuc9pgc9s8qqaq=dirpe0xb9q8qiLsFr0=vr0=vr0dc8meaabaqaciaacaGaaeqabaqabeGadaaakeaacuWGKbazgaqeamaaBaaaleaacqWGnbqtcqGGSaaliiGacqWFXoqyaeqaaaaa@31EA@ is the one which provides the best separation of classes in terms of the Manhattan distance:

αM∗=arg⁡max⁡α(d¯M,α)
 MathType@MTEF@5@5@+=feaafiart1ev1aaatCvAUfKttLearuWrP9MDH5MBPbIqV92AaeXatLxBI9gBaebbnrfifHhDYfgasaacH8akY=wiFfYdH8Gipec8Eeeu0xXdbba9frFj0=OqFfea0dXdd9vqai=hGuQ8kuc9pgc9s8qqaq=dirpe0xb9q8qiLsFr0=vr0=vr0dc8meaabaqaciaacaGaaeqabaqabeGadaaakeaaiiGacqWFXoqydaqhaaWcbaGaemyta0eabaGaey4fIOcaaOGaeyypa0ZaaCbeaeaacyGGHbqycqGGYbGCcqGGNbWzcyGGTbqBcqGGHbqycqGG4baEaSqaaiab=f7aHbqabaGcdaqadaqaaiqbdsgaKzaaraWaaSbaaSqaaiabd2eanjabcYcaSiab=f7aHbqabaaakiaawIcacaGLPaaaaaa@4277@

If there is more than one value of *α *satisfying equations (9) or (12), the mean among these values is taken as αE∗
 MathType@MTEF@5@5@+=feaafiart1ev1aaatCvAUfKttLearuWrP9MDH5MBPbIqV92AaeXatLxBI9gBaebbnrfifHhDYfgasaacH8akY=wiFfYdH8Gipec8Eeeu0xXdbba9frFj0=OqFfea0dXdd9vqai=hGuQ8kuc9pgc9s8qqaq=dirpe0xb9q8qiLsFr0=vr0=vr0dc8meaabaqaciaacaGaaeqabaqabeGadaaakeaaiiGacqWFXoqydaqhaaWcbaGaemyraueabaGaey4fIOcaaaaa@3081@ or αM∗
 MathType@MTEF@5@5@+=feaafiart1ev1aaatCvAUfKttLearuWrP9MDH5MBPbIqV92AaeXatLxBI9gBaebbnrfifHhDYfgasaacH8akY=wiFfYdH8Gipec8Eeeu0xXdbba9frFj0=OqFfea0dXdd9vqai=hGuQ8kuc9pgc9s8qqaq=dirpe0xb9q8qiLsFr0=vr0=vr0dc8meaabaqaciaacaGaaeqabaqabeGadaaakeaaiiGacqWFXoqydaqhaaWcbaGaemyta0eabaGaey4fIOcaaaaa@3091@. Since these values are generally observed to be adjacent to each other, taking the mean will still provide a good picture of how the DDP affects separation of classes.

**Figure 1:**
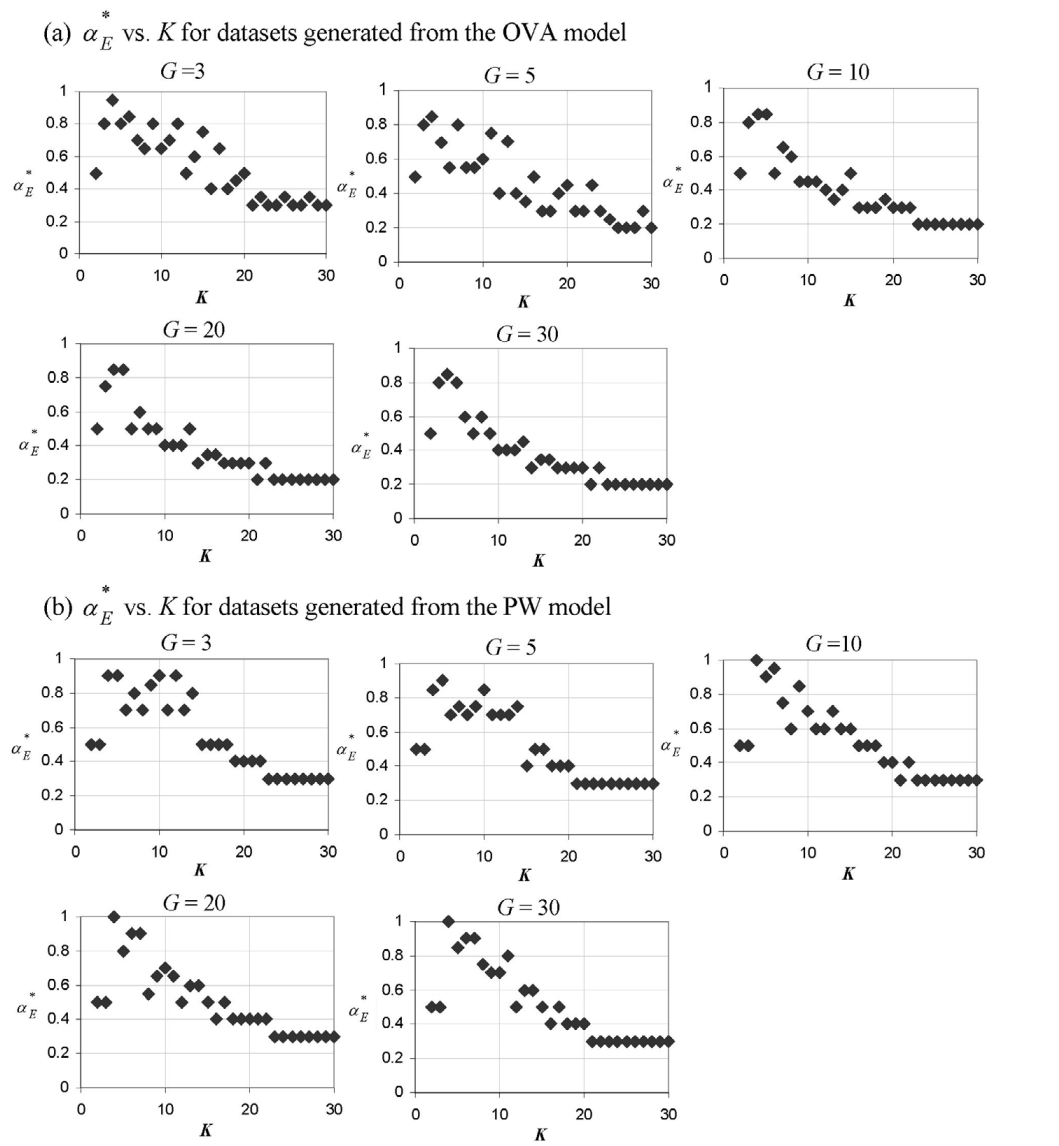
**Plots of **αE∗
 MathType@MTEF@5@5@+=feaafiart1ev1aaatCvAUfKttLearuWrP9MDH5MBPbIqV92AaeXatLxBI9gBaebbnrfifHhDYfgasaacH8akY=wiFfYdH8Gipec8Eeeu0xXdbba9frFj0=OqFfea0dXdd9vqai=hGuQ8kuc9pgc9s8qqaq=dirpe0xb9q8qiLsFr0=vr0=vr0dc8meaabaqaciaacaGaaeqabaqabeGadaaakeaaiiGacqWFXoqydaqhaaWcbaGaemyraueabaGaey4fIOcaaaaa@3081@** vs. *K***. The DDP producing the optimal separation of classes as measured using the Euclidean distance, αE∗
 MathType@MTEF@5@5@+=feaafiart1ev1aaatCvAUfKttLearuWrP9MDH5MBPbIqV92AaeXatLxBI9gBaebbnrfifHhDYfgasaacH8akY=wiFfYdH8Gipec8Eeeu0xXdbba9frFj0=OqFfea0dXdd9vqai=hGuQ8kuc9pgc9s8qqaq=dirpe0xb9q8qiLsFr0=vr0=vr0dc8meaabaqaciaacaGaaeqabaqabeGadaaakeaaiiGacqWFXoqydaqhaaWcbaGaemyraueabaGaey4fIOcaaaaa@3081@, as a function of *K *for toy datasets generated from (a) the OVA model and (b) the PW model. Each of the five panels in (a) and (b) represents a plot from toy datasets generated using a different value of *G *(a parameter which denotes the number of genes in each group of marker genes and is set during the generation of the toy datasets).

Figure 1 shows that the number of classes, *K*, influences the value of αE∗
 MathType@MTEF@5@5@+=feaafiart1ev1aaatCvAUfKttLearuWrP9MDH5MBPbIqV92AaeXatLxBI9gBaebbnrfifHhDYfgasaacH8akY=wiFfYdH8Gipec8Eeeu0xXdbba9frFj0=OqFfea0dXdd9vqai=hGuQ8kuc9pgc9s8qqaq=dirpe0xb9q8qiLsFr0=vr0=vr0dc8meaabaqaciaacaGaaeqabaqabeGadaaakeaaiiGacqWFXoqydaqhaaWcbaGaemyraueabaGaey4fIOcaaaaa@3081@, regardless of the value set to *G*. Larger *G *tends to produce a more distinct αE∗
 MathType@MTEF@5@5@+=feaafiart1ev1aaatCvAUfKttLearuWrP9MDH5MBPbIqV92AaeXatLxBI9gBaebbnrfifHhDYfgasaacH8akY=wiFfYdH8Gipec8Eeeu0xXdbba9frFj0=OqFfea0dXdd9vqai=hGuQ8kuc9pgc9s8qqaq=dirpe0xb9q8qiLsFr0=vr0=vr0dc8meaabaqaciaacaGaaeqabaqabeGadaaakeaaiiGacqWFXoqydaqhaaWcbaGaemyraueabaGaey4fIOcaaaaa@3081@ - *K *plot. As *K *increases beyond 20, αE∗
 MathType@MTEF@5@5@+=feaafiart1ev1aaatCvAUfKttLearuWrP9MDH5MBPbIqV92AaeXatLxBI9gBaebbnrfifHhDYfgasaacH8akY=wiFfYdH8Gipec8Eeeu0xXdbba9frFj0=OqFfea0dXdd9vqai=hGuQ8kuc9pgc9s8qqaq=dirpe0xb9q8qiLsFr0=vr0=vr0dc8meaabaqaciaacaGaaeqabaqabeGadaaakeaaiiGacqWFXoqydaqhaaWcbaGaemyraueabaGaey4fIOcaaaaa@3081@ settles to a smaller value (around 0.2) for OVA toy datasets than for PW toy datasets (around 0.3). This is due to the difference in the rate of decline, which is greater for OVA toy datasets than for PW toy datasets. Regardless of the model type or the value the model parameter, *G*, is set to, the number of classes in the dataset undoubtedly affects the value of the DDP which produces the best separation of classes in terms of the Euclidean distance.

**Figure 2:**
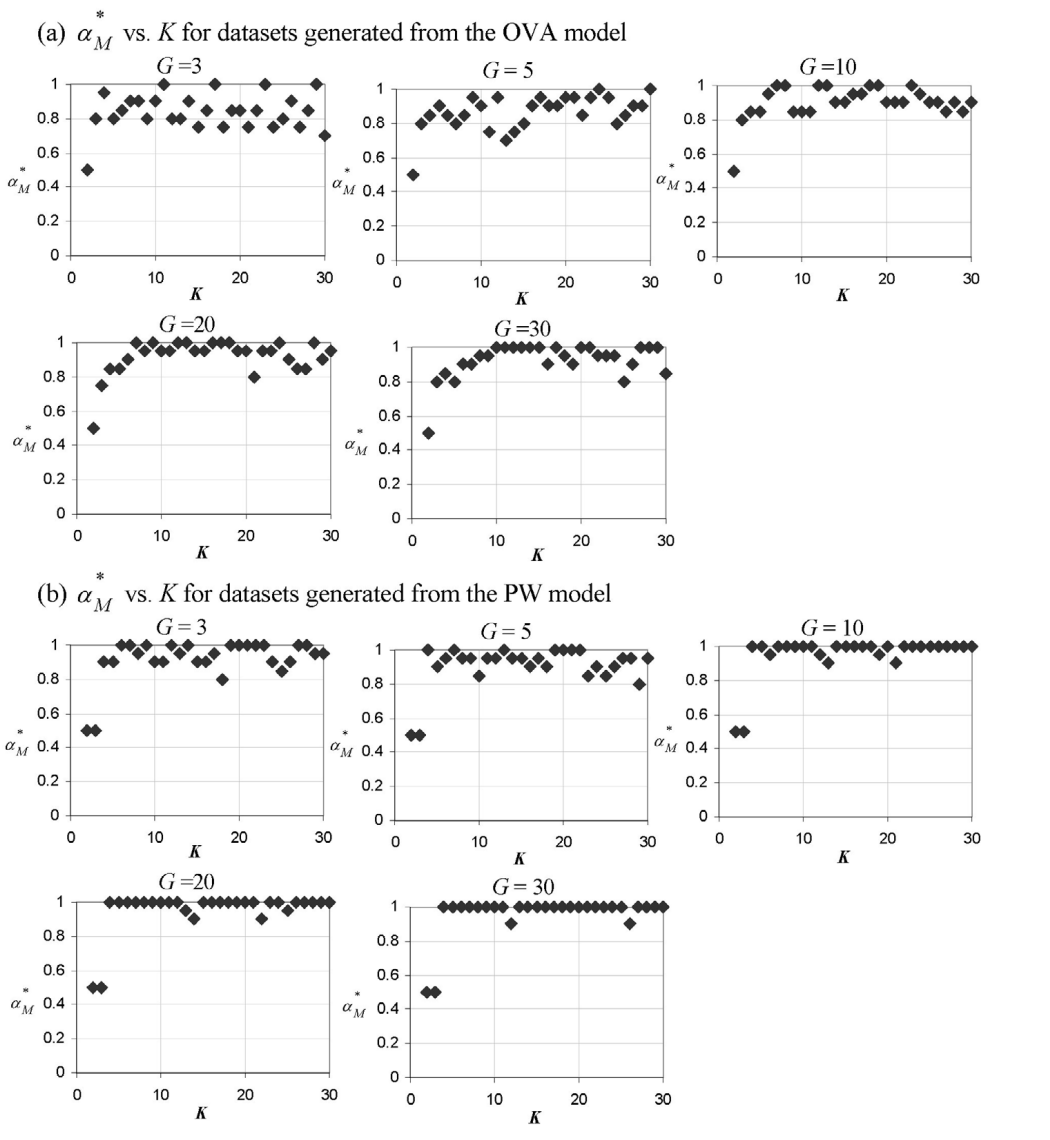
**Plots of **αM∗
 MathType@MTEF@5@5@+=feaafiart1ev1aaatCvAUfKttLearuWrP9MDH5MBPbIqV92AaeXatLxBI9gBaebbnrfifHhDYfgasaacH8akY=wiFfYdH8Gipec8Eeeu0xXdbba9frFj0=OqFfea0dXdd9vqai=hGuQ8kuc9pgc9s8qqaq=dirpe0xb9q8qiLsFr0=vr0=vr0dc8meaabaqaciaacaGaaeqabaqabeGadaaakeaaiiGacqWFXoqydaqhaaWcbaGaemyta0eabaGaey4fIOcaaaaa@3091@** vs. *K***. The DDP producing the optimal separation of classes as measured using the Manhattan distance, αM∗
 MathType@MTEF@5@5@+=feaafiart1ev1aaatCvAUfKttLearuWrP9MDH5MBPbIqV92AaeXatLxBI9gBaebbnrfifHhDYfgasaacH8akY=wiFfYdH8Gipec8Eeeu0xXdbba9frFj0=OqFfea0dXdd9vqai=hGuQ8kuc9pgc9s8qqaq=dirpe0xb9q8qiLsFr0=vr0=vr0dc8meaabaqaciaacaGaaeqabaqabeGadaaakeaaiiGacqWFXoqydaqhaaWcbaGaemyta0eabaGaey4fIOcaaaaa@3091@, as a function of *K *for toy datasets generated from (a) the OVA model and (b) the PW model. Each of the five panels in (a) and (b) represents a plot from toy datasets generated using a different value of *G *(a parameter which denotes the number of genes in each group of marker genes and is set during the generation of the toy datasets).

Conversely, we find this to be untrue in terms of the Manhattan distance (Figure 2). Regardless of the number of classes, *K*, the value of αM∗
 MathType@MTEF@5@5@+=feaafiart1ev1aaatCvAUfKttLearuWrP9MDH5MBPbIqV92AaeXatLxBI9gBaebbnrfifHhDYfgasaacH8akY=wiFfYdH8Gipec8Eeeu0xXdbba9frFj0=OqFfea0dXdd9vqai=hGuQ8kuc9pgc9s8qqaq=dirpe0xb9q8qiLsFr0=vr0=vr0dc8meaabaqaciaacaGaaeqabaqabeGadaaakeaaiiGacqWFXoqydaqhaaWcbaGaemyta0eabaGaey4fIOcaaaaa@3091@ remains around the range [0.8,1], near the DDP value for rank-based selection. (See the Background section for details on the significance of the values of the DDP.)

### Principal component analysis (PCA)

PCA linearly transforms the data such that the greatest amount of variance among samples comes to lie along the axis representing the first principal component (PC). Similarly, the second PC contains the second largest variance among samples, and so on. An important property of the PCs is that a PC is always orthogonal to the adjacent PC.

In addition to analyzing the predictor sets in the original projection, we investigate the characteristics of the predictor sets after transformation by PCA. In the original form, the data are characterized along axes representing members of the predictor set (original feature space). After transformation by PCA, data are characterized along axes representing the PCs derived from the members of the predictor set (PCA-transformed space or PC space).

The input data matrix is never mean-centered throughout the transformation procedures – this is to enable comparisons in terms of distance metrics between data in original feature space and data in PC space later in this study. (For instance, in this manner, the Euclidean distance remains constant in both original feature space and PC space.) The sole effect of not mean-centering the dataset is that the first PC will span the variance characterized by the overall distance of the dataset from the origin [15]. In case of our models (OVA and PW), marker genes contain non-zero class mean for each of the classes (OVA model) or non-zero class means for each of the pairs of classes (PW model) that they mark, and zero class means for all other classes. Thus for both models, even without mean-centering, the variance contained by the first PC will still be variance among classes, because for both models, the distance of a data point (a sample) from the origin as measured by each gene is actually characterized by the class of that data point.

The main use of PCA in this case is to rotate the data from the original sets of axes (represented by the members of the predictor set) so that the data are now projected along new sets of axes (represented by the PCs) which are orthogonal and hence minimally correlated to each other. In this study, PCA is conducted only on the members of the predictor set, not on the whole dataset. The reason we apply PCA in this manner is to expand on the finding in [3] which we discuss in the beginning of the "Separation of classes" subsection. Therefore, each of the PCs in this study contains information only from the predictor set, and never from any gene which is not a member of the predictor set.

### Antiredundancy of PCA-transformed predictor sets

Let us denote the antiredundancy of predictor set *S*_*α *_after transformation by PCA as U′Sα
 MathType@MTEF@5@5@+=feaafiart1ev1aaatCvAUfKttLearuWrP9MDH5MBPbIqV92AaeXatLxBI9gBaebbnrfifHhDYfgasaacH8akY=wiFfYdH8Gipec8Eeeu0xXdbba9frFj0=OqFfea0dXdd9vqai=hGuQ8kuc9pgc9s8qqaq=dirpe0xb9q8qiLsFr0=vr0=vr0dc8meaabaqaciaacaGaaeqabaqabeGadaaakeaacuWGvbqvgaqbamaaBaaaleaacqWGtbWudaWgaaadbaacciGae8xSdegabeaaaSqabaaaaa@3124@. The value of the DDP giving the largest antiredundancy in PC space is defined as follows:

αU∗=arg⁡max⁡α(U′Sα)
 MathType@MTEF@5@5@+=feaafiart1ev1aaatCvAUfKttLearuWrP9MDH5MBPbIqV92AaeXatLxBI9gBaebbnrfifHhDYfgasaacH8akY=wiFfYdH8Gipec8Eeeu0xXdbba9frFj0=OqFfea0dXdd9vqai=hGuQ8kuc9pgc9s8qqaq=dirpe0xb9q8qiLsFr0=vr0=vr0dc8meaabaqaciaacaGaaeqabaqabeGadaaakeaaiiGacqWFXoqydaqhaaWcbaGaemyvaufabaGaey4fIOcaaOGaeyypa0ZaaCbeaeaacyGGHbqycqGGYbGCcqGGNbWzcyGGTbqBcqGGHbqycqGG4baEaSqaaiab=f7aHbqabaGcdaqadaqaaiqbdwfavzaafaWaaSbaaSqaaiabdofatnaaBaaameaacqWFXoqyaeqaaaWcbeaaaOGaayjkaiaawMcaaaaa@41C1@

**Figure 3:**
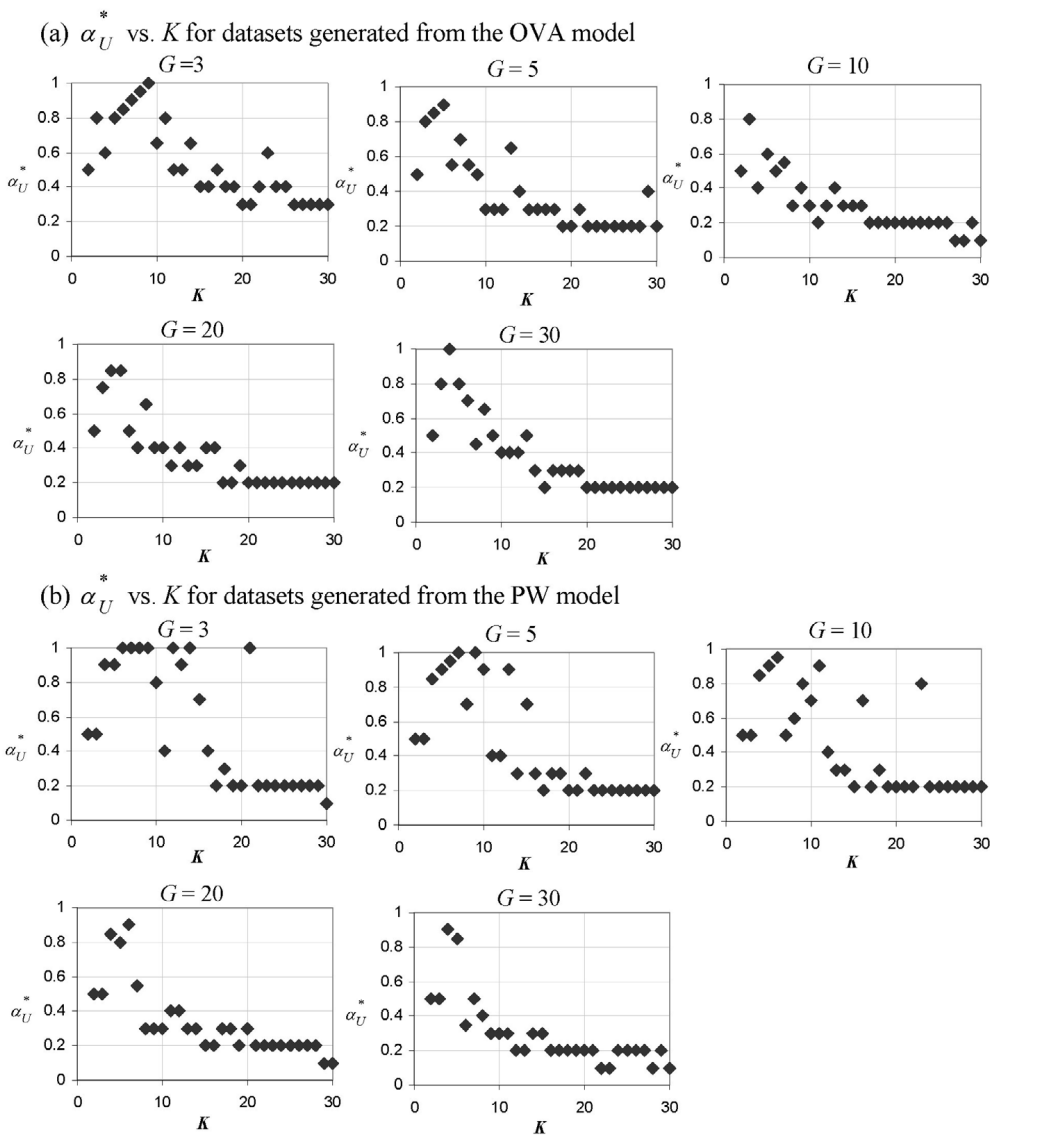
**Plots of **αU∗
 MathType@MTEF@5@5@+=feaafiart1ev1aaatCvAUfKttLearuWrP9MDH5MBPbIqV92AaeXatLxBI9gBaebbnrfifHhDYfgasaacH8akY=wiFfYdH8Gipec8Eeeu0xXdbba9frFj0=OqFfea0dXdd9vqai=hGuQ8kuc9pgc9s8qqaq=dirpe0xb9q8qiLsFr0=vr0=vr0dc8meaabaqaciaacaGaaeqabaqabeGadaaakeaaiiGacqWFXoqydaqhaaWcbaGaemyvaufabaGaey4fIOcaaaaa@30A1@** vs. *K***. The DDP producing the optimal antiredundancy as measured in PC space, αU∗
 MathType@MTEF@5@5@+=feaafiart1ev1aaatCvAUfKttLearuWrP9MDH5MBPbIqV92AaeXatLxBI9gBaebbnrfifHhDYfgasaacH8akY=wiFfYdH8Gipec8Eeeu0xXdbba9frFj0=OqFfea0dXdd9vqai=hGuQ8kuc9pgc9s8qqaq=dirpe0xb9q8qiLsFr0=vr0=vr0dc8meaabaqaciaacaGaaeqabaqabeGadaaakeaaiiGacqWFXoqydaqhaaWcbaGaemyvaufabaGaey4fIOcaaaaa@30A1@, as a function of *K *for toy datasets generated from (a) the OVA model and (b) the PW model. Each of the five panels in (a) and (b) represents a plot from toy datasets generated using a different value of *G *(a parameter which denotes the number of genes in each group of marker genes and is set during the generation of the toy datasets).

For untransformed predictor sets, the value of the DDP satisfying the expression USα
 MathType@MTEF@5@5@+=feaafiart1ev1aaatCvAUfKttLearuWrP9MDH5MBPbIqV92AaeXatLxBI9gBaebbnrfifHhDYfgasaacH8akY=wiFfYdH8Gipec8Eeeu0xXdbba9frFj0=OqFfea0dXdd9vqai=hGuQ8kuc9pgc9s8qqaq=dirpe0xb9q8qiLsFr0=vr0=vr0dc8meaabaqaciaacaGaaeqabaqabeGadaaakeaacqWGvbqvdaWgaaWcbaGaem4uam1aaSbaaWqaaGGaciab=f7aHbqabaaaleqaaaaa@3118@ is naturally 0. However, this is not so for PCA-transformed predictor sets. In Figure 3, we observe that *K *has a similar effect on αU∗
 MathType@MTEF@5@5@+=feaafiart1ev1aaatCvAUfKttLearuWrP9MDH5MBPbIqV92AaeXatLxBI9gBaebbnrfifHhDYfgasaacH8akY=wiFfYdH8Gipec8Eeeu0xXdbba9frFj0=OqFfea0dXdd9vqai=hGuQ8kuc9pgc9s8qqaq=dirpe0xb9q8qiLsFr0=vr0=vr0dc8meaabaqaciaacaGaaeqabaqabeGadaaakeaaiiGacqWFXoqydaqhaaWcbaGaemyvaufabaGaey4fIOcaaaaa@30A1@ as it has on αE∗
 MathType@MTEF@5@5@+=feaafiart1ev1aaatCvAUfKttLearuWrP9MDH5MBPbIqV92AaeXatLxBI9gBaebbnrfifHhDYfgasaacH8akY=wiFfYdH8Gipec8Eeeu0xXdbba9frFj0=OqFfea0dXdd9vqai=hGuQ8kuc9pgc9s8qqaq=dirpe0xb9q8qiLsFr0=vr0=vr0dc8meaabaqaciaacaGaaeqabaqabeGadaaakeaaiiGacqWFXoqydaqhaaWcbaGaemyraueabaGaey4fIOcaaaaa@3081@. As *K *increases, the value of the DDP needed to produce a predictor set with the highest U′Sα
 MathType@MTEF@5@5@+=feaafiart1ev1aaatCvAUfKttLearuWrP9MDH5MBPbIqV92AaeXatLxBI9gBaebbnrfifHhDYfgasaacH8akY=wiFfYdH8Gipec8Eeeu0xXdbba9frFj0=OqFfea0dXdd9vqai=hGuQ8kuc9pgc9s8qqaq=dirpe0xb9q8qiLsFr0=vr0=vr0dc8meaabaqaciaacaGaaeqabaqabeGadaaakeaacuWGvbqvgaqbamaaBaaaleaacqWGtbWudaWgaaadbaacciGae8xSdegabeaaaSqabaaaaa@3124@ decreases in an exponential-like manner. Also, similar to the case of αE∗
 MathType@MTEF@5@5@+=feaafiart1ev1aaatCvAUfKttLearuWrP9MDH5MBPbIqV92AaeXatLxBI9gBaebbnrfifHhDYfgasaacH8akY=wiFfYdH8Gipec8Eeeu0xXdbba9frFj0=OqFfea0dXdd9vqai=hGuQ8kuc9pgc9s8qqaq=dirpe0xb9q8qiLsFr0=vr0=vr0dc8meaabaqaciaacaGaaeqabaqabeGadaaakeaaiiGacqWFXoqydaqhaaWcbaGaemyraueabaGaey4fIOcaaaaa@3081@, larger values of *G *generate better-defined αU∗
 MathType@MTEF@5@5@+=feaafiart1ev1aaatCvAUfKttLearuWrP9MDH5MBPbIqV92AaeXatLxBI9gBaebbnrfifHhDYfgasaacH8akY=wiFfYdH8Gipec8Eeeu0xXdbba9frFj0=OqFfea0dXdd9vqai=hGuQ8kuc9pgc9s8qqaq=dirpe0xb9q8qiLsFr0=vr0=vr0dc8meaabaqaciaacaGaaeqabaqabeGadaaakeaaiiGacqWFXoqydaqhaaWcbaGaemyvaufabaGaey4fIOcaaaaa@30A1@ - *K *curves. Model type (OVA or PW) does not affect the shape of the αU∗
 MathType@MTEF@5@5@+=feaafiart1ev1aaatCvAUfKttLearuWrP9MDH5MBPbIqV92AaeXatLxBI9gBaebbnrfifHhDYfgasaacH8akY=wiFfYdH8Gipec8Eeeu0xXdbba9frFj0=OqFfea0dXdd9vqai=hGuQ8kuc9pgc9s8qqaq=dirpe0xb9q8qiLsFr0=vr0=vr0dc8meaabaqaciaacaGaaeqabaqabeGadaaakeaaiiGacqWFXoqydaqhaaWcbaGaemyvaufabaGaey4fIOcaaaaa@30A1@ - *K *plot as much as it does the shape of the αE∗
 MathType@MTEF@5@5@+=feaafiart1ev1aaatCvAUfKttLearuWrP9MDH5MBPbIqV92AaeXatLxBI9gBaebbnrfifHhDYfgasaacH8akY=wiFfYdH8Gipec8Eeeu0xXdbba9frFj0=OqFfea0dXdd9vqai=hGuQ8kuc9pgc9s8qqaq=dirpe0xb9q8qiLsFr0=vr0=vr0dc8meaabaqaciaacaGaaeqabaqabeGadaaakeaaiiGacqWFXoqydaqhaaWcbaGaemyraueabaGaey4fIOcaaaaa@3081@ - *K *plot. The converging value of αU∗
 MathType@MTEF@5@5@+=feaafiart1ev1aaatCvAUfKttLearuWrP9MDH5MBPbIqV92AaeXatLxBI9gBaebbnrfifHhDYfgasaacH8akY=wiFfYdH8Gipec8Eeeu0xXdbba9frFj0=OqFfea0dXdd9vqai=hGuQ8kuc9pgc9s8qqaq=dirpe0xb9q8qiLsFr0=vr0=vr0dc8meaabaqaciaacaGaaeqabaqabeGadaaakeaaiiGacqWFXoqydaqhaaWcbaGaemyvaufabaGaey4fIOcaaaaa@30A1@ is around 0.2 for both models.

### Separation of classes in PCA-transformed predictor sets

The Euclidean distance remains the same whether the predictor sets have been transformed by PCA or not; hence we do not repeat the analysis described in the previous subsection (on separation of classes) for PCA-transformed predictor sets. The Manhattan distance, however, *is *affected by the transformation. The procedures involved in computing the DDP value leading to the best separation of classes are the same as in case of the untransformed predictor sets described in the previous subsection (on separation of classes). To distinguish between the DDP value associated with untransformed predictor sets and the DDP value associated with PCA-transformed predictor sets, we will denote the latter as αMP∗
 MathType@MTEF@5@5@+=feaafiart1ev1aaatCvAUfKttLearuWrP9MDH5MBPbIqV92AaeXatLxBI9gBaebbnrfifHhDYfgasaacH8akY=wiFfYdH8Gipec8Eeeu0xXdbba9frFj0=OqFfea0dXdd9vqai=hGuQ8kuc9pgc9s8qqaq=dirpe0xb9q8qiLsFr0=vr0=vr0dc8meaabaqaciaacaGaaeqabaqabeGadaaakeaaiiGacqWFXoqydaqhaaWcbaGaemyta0KaemiuaafabaGaey4fIOcaaaaa@31BA@. Similarly, separation of classes in PC space in terms of the Manhattan distance as measured by a predictor set found using the DDP value of *α*, *S*_*α*_, is denoted as d¯MP,α
 MathType@MTEF@5@5@+=feaafiart1ev1aaatCvAUfKttLearuWrP9MDH5MBPbIqV92AaeXatLxBI9gBaebbnrfifHhDYfgasaacH8akY=wiFfYdH8Gipec8Eeeu0xXdbba9frFj0=OqFfea0dXdd9vqai=hGuQ8kuc9pgc9s8qqaq=dirpe0xb9q8qiLsFr0=vr0=vr0dc8meaabaqaciaacaGaaeqabaqabeGadaaakeaacuWGKbazgaqeamaaBaaaleaacqWGnbqtcqWGqbaucqGGSaaliiGacqWFXoqyaeqaaaaa@3313@.

**Figure 4:**
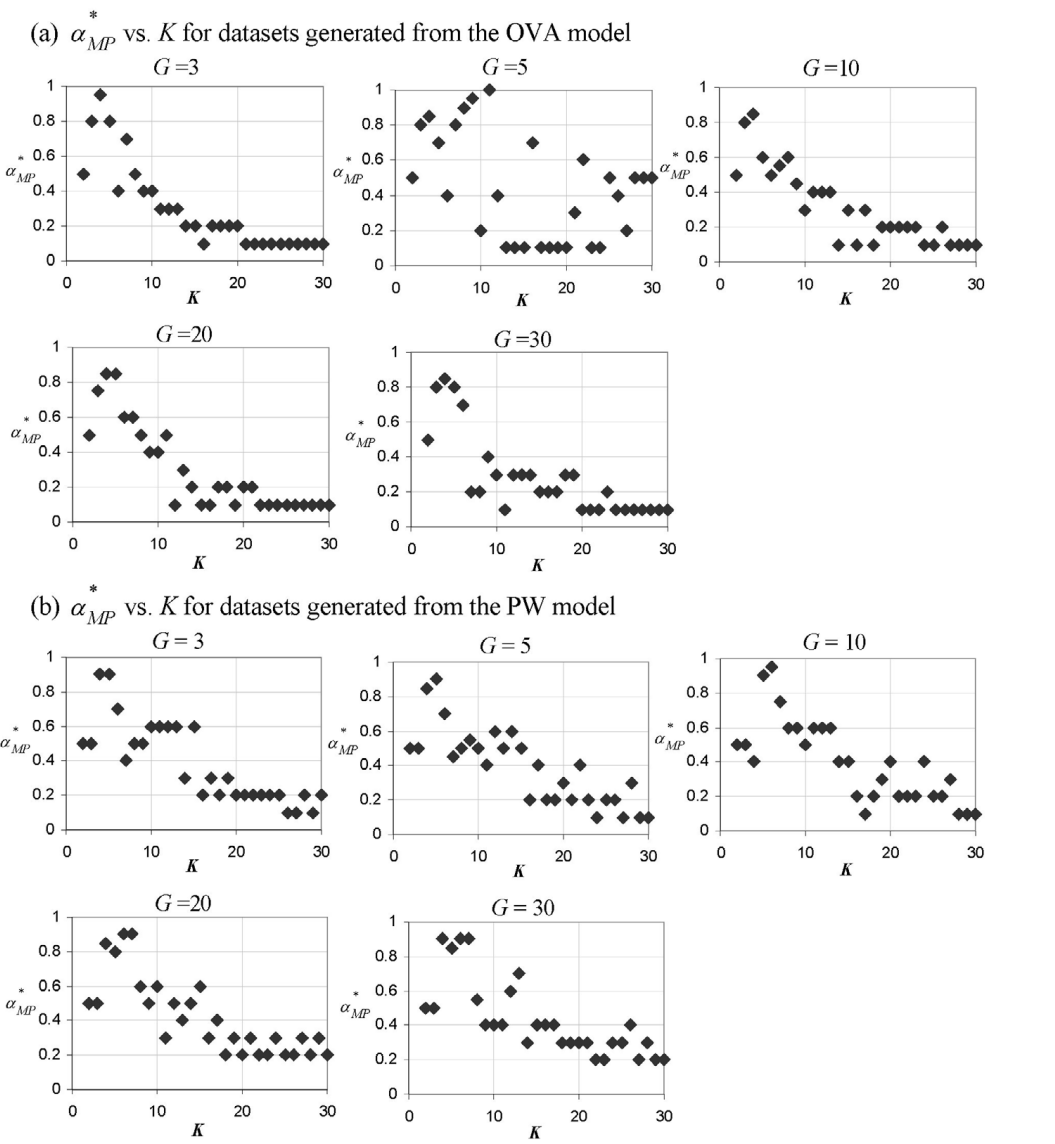
**Plots of **αMP∗
 MathType@MTEF@5@5@+=feaafiart1ev1aaatCvAUfKttLearuWrP9MDH5MBPbIqV92AaeXatLxBI9gBaebbnrfifHhDYfgasaacH8akY=wiFfYdH8Gipec8Eeeu0xXdbba9frFj0=OqFfea0dXdd9vqai=hGuQ8kuc9pgc9s8qqaq=dirpe0xb9q8qiLsFr0=vr0=vr0dc8meaabaqaciaacaGaaeqabaqabeGadaaakeaaiiGacqWFXoqydaqhaaWcbaGaemyta0KaemiuaafabaGaey4fIOcaaaaa@31BA@** vs. *K***. The DDP producing the optimal separation of classes as measured using the Manhattan distance in PC space, αMP∗
 MathType@MTEF@5@5@+=feaafiart1ev1aaatCvAUfKttLearuWrP9MDH5MBPbIqV92AaeXatLxBI9gBaebbnrfifHhDYfgasaacH8akY=wiFfYdH8Gipec8Eeeu0xXdbba9frFj0=OqFfea0dXdd9vqai=hGuQ8kuc9pgc9s8qqaq=dirpe0xb9q8qiLsFr0=vr0=vr0dc8meaabaqaciaacaGaaeqabaqabeGadaaakeaaiiGacqWFXoqydaqhaaWcbaGaemyta0KaemiuaafabaGaey4fIOcaaaaa@31BA@, as a function of *K *for toy datasets generated from (a) the OVA model and (b) the PW model. Each of the five panels in (a) and (b) represents a plot from toy datasets generated using a different value of *G *(a parameter which denotes the number of genes in each group of marker genes and is set during the generation of the toy datasets).

Although we have seen from Figure 2 that *K *has no effect on αM∗
 MathType@MTEF@5@5@+=feaafiart1ev1aaatCvAUfKttLearuWrP9MDH5MBPbIqV92AaeXatLxBI9gBaebbnrfifHhDYfgasaacH8akY=wiFfYdH8Gipec8Eeeu0xXdbba9frFj0=OqFfea0dXdd9vqai=hGuQ8kuc9pgc9s8qqaq=dirpe0xb9q8qiLsFr0=vr0=vr0dc8meaabaqaciaacaGaaeqabaqabeGadaaakeaaiiGacqWFXoqydaqhaaWcbaGaemyta0eabaGaey4fIOcaaaaa@3091@, Figure 4 clearly demonstrates that the increase of *K *produces an exponential-like decrease in αMP∗
 MathType@MTEF@5@5@+=feaafiart1ev1aaatCvAUfKttLearuWrP9MDH5MBPbIqV92AaeXatLxBI9gBaebbnrfifHhDYfgasaacH8akY=wiFfYdH8Gipec8Eeeu0xXdbba9frFj0=OqFfea0dXdd9vqai=hGuQ8kuc9pgc9s8qqaq=dirpe0xb9q8qiLsFr0=vr0=vr0dc8meaabaqaciaacaGaaeqabaqabeGadaaakeaaiiGacqWFXoqydaqhaaWcbaGaemyta0KaemiuaafabaGaey4fIOcaaaaa@31BA@. The plots in Figure 4 are generally more clear-cut for toy datasets based on the OVA model than those based on the PW model, especially when *G *is greater than 5. As in the case of αE∗
 MathType@MTEF@5@5@+=feaafiart1ev1aaatCvAUfKttLearuWrP9MDH5MBPbIqV92AaeXatLxBI9gBaebbnrfifHhDYfgasaacH8akY=wiFfYdH8Gipec8Eeeu0xXdbba9frFj0=OqFfea0dXdd9vqai=hGuQ8kuc9pgc9s8qqaq=dirpe0xb9q8qiLsFr0=vr0=vr0dc8meaabaqaciaacaGaaeqabaqabeGadaaakeaaiiGacqWFXoqydaqhaaWcbaGaemyraueabaGaey4fIOcaaaaa@3081@, the decline rate is greater for toy datasets generated from the OVA model than those from the PW model. The converging value of αMP∗
 MathType@MTEF@5@5@+=feaafiart1ev1aaatCvAUfKttLearuWrP9MDH5MBPbIqV92AaeXatLxBI9gBaebbnrfifHhDYfgasaacH8akY=wiFfYdH8Gipec8Eeeu0xXdbba9frFj0=OqFfea0dXdd9vqai=hGuQ8kuc9pgc9s8qqaq=dirpe0xb9q8qiLsFr0=vr0=vr0dc8meaabaqaciaacaGaaeqabaqabeGadaaakeaaiiGacqWFXoqydaqhaaWcbaGaemyta0KaemiuaafabaGaey4fIOcaaaaa@31BA@ as *K *increases beyond 20 is 0.1 for all *G *in case of the OVA model. In case of the PW model it lies between 0.1 and 0.2 for *G *= 3, 5, 10 and between 0.2 and 0.3 for *G *= 20, 30.

Indeed, the observation regarding αMP∗
 MathType@MTEF@5@5@+=feaafiart1ev1aaatCvAUfKttLearuWrP9MDH5MBPbIqV92AaeXatLxBI9gBaebbnrfifHhDYfgasaacH8akY=wiFfYdH8Gipec8Eeeu0xXdbba9frFj0=OqFfea0dXdd9vqai=hGuQ8kuc9pgc9s8qqaq=dirpe0xb9q8qiLsFr0=vr0=vr0dc8meaabaqaciaacaGaaeqabaqabeGadaaakeaaiiGacqWFXoqydaqhaaWcbaGaemyta0KaemiuaafabaGaey4fIOcaaaaa@31BA@ provides the link between the study in [3] and the DDP concept. In almost all cases, a predictor set which is obtained using the DDP value of αMP∗
 MathType@MTEF@5@5@+=feaafiart1ev1aaatCvAUfKttLearuWrP9MDH5MBPbIqV92AaeXatLxBI9gBaebbnrfifHhDYfgasaacH8akY=wiFfYdH8Gipec8Eeeu0xXdbba9frFj0=OqFfea0dXdd9vqai=hGuQ8kuc9pgc9s8qqaq=dirpe0xb9q8qiLsFr0=vr0=vr0dc8meaabaqaciaacaGaaeqabaqabeGadaaakeaaiiGacqWFXoqydaqhaaWcbaGaemyta0KaemiuaafabaGaey4fIOcaaaaa@31BA@ shows enhanced separation of classes in PC space compared to separation of classes in the original feature space (measured using the Manhattan distance) – a finding which is reflected in that study described earlier in the beginning of the "Separation of classes" subsection. Therefore at the optimal value of the DDP, separation of classes as measured using the Manhattan distance in PC space is maximized because of this enhancement.

### Summary of analyses

We have found that as *K *increases, three parameters decrease in an exponential-like manner:

• the value of the DDP producing the best separation of classes in terms of the Euclidean distance;

• the value of the DDP producing the highest antiredundancy in PC space; and

• the value of the DDP producing the best separation of classes in terms of the Manhattan distance in PC space.

We have shown that regardless of the model type (OVA or PW) or the value set to the model parameter, *G *(3, 5, 10, 20, or 30), each of these three characteristics can be optimized by choosing the right value of the DDP, and that this value, in turn, is determined by the number of classes in the dataset.

### Investigating the imbalance of class means in real-life datasets

Before reapplying the analyses to real-life datasets, we investigate how close conditions in real-life datasets match those of toy datasets. We have mentioned in the section on the generation of toy datasets that imbalance in terms of class means among classes is prevalent in highly multiclass microarray datasets. Investigation is conducted on whole datasets (no splitting) in this case. For class *k*, we choose the class mean with the greatest absolute value (equivalent to the absolute value of *μ*_(*g *- 1)*K*+*k*,*k *_from equation (4) or μ(g−1)⋅(K2)+q,cb,q
 MathType@MTEF@5@5@+=feaafiart1ev1aaatCvAUfKttLearuWrP9MDH5MBPbIqV92AaeXatLxBI9gBaebbnrfifHhDYfgasaacH8akY=wiFfYdH8Gipec8Eeeu0xXdbba9frFj0=OqFfea0dXdd9vqai=hGuQ8kuc9pgc9s8qqaq=dirpe0xb9q8qiLsFr0=vr0=vr0dc8meaabaqaciaacaGaaeqabaqabeGadaaakeaaiiGacqWF8oqBdaWgaaWcbaGaeiikaGIaem4zaCMaeyOeI0IaeGymaeJaeiykaKIaeyyXIC9aaeWaaeaafaqabeGabaaabaGaem4saSeabaGaeGOmaidaaaGaayjkaiaawMcaaiabgUcaRiabdghaXjabcYcaSiabdogaJnaaBaaameaacqWGIbGycqGGSaalcqWGXbqCaeqaaaWcbeaaaaa@41B8@ from equation (5) at *g *= 1) among all *N *class means:

x¯0,k=max⁡i=1,2,...,N(|x¯i,k|)
 MathType@MTEF@5@5@+=feaafiart1ev1aaatCvAUfKttLearuWrP9MDH5MBPbIqV92AaeXatLxBI9gBaebbnrfifHhDYfgasaacH8akY=wiFfYdH8Gipec8Eeeu0xXdbba9frFj0=OqFfea0dXdd9vqai=hGuQ8kuc9pgc9s8qqaq=dirpe0xb9q8qiLsFr0=vr0=vr0dc8meaabaqaciaacaGaaeqabaqabeGadaaakeaacuWG4baEgaqeamaaBaaaleaacqaIWaamcqGGSaalcqWGRbWAaeqaaOGaeyypa0ZaaCbeaeaacyGGTbqBcqGGHbqycqGG4baEaSqaaiabdMgaPjabg2da9iabigdaXiabcYcaSiabikdaYiabcYcaSiabc6caUiabc6caUiabc6caUiabcYcaSiabd6eaobqabaGcdaqadaqaamaaemaabaGafmiEaGNbaebadaWgaaWcbaGaemyAaKMaeiilaWIaem4AaSgabeaaaOGaay5bSlaawIa7aaGaayjkaiaawMcaaaaa@4BCF@

Next, to illustrate the imbalance among classes in terms of class means, we compute the range of class means:

R(x¯0,k)=max⁡k(x¯0,k)−min⁡k(x¯0,k)
 MathType@MTEF@5@5@+=feaafiart1ev1aaatCvAUfKttLearuWrP9MDH5MBPbIqV92AaeXatLxBI9gBaebbnrfifHhDYfgasaacH8akY=wiFfYdH8Gipec8Eeeu0xXdbba9frFj0=OqFfea0dXdd9vqai=hGuQ8kuc9pgc9s8qqaq=dirpe0xb9q8qiLsFr0=vr0=vr0dc8meaabaqaciaacaGaaeqabaqabeGadaaakeaacqWGsbGudaqadaqaaiqbdIha4zaaraWaaSbaaSqaaiabicdaWiabcYcaSiabdUgaRbqabaaakiaawIcacaGLPaaacqGH9aqpdaWfqaqaaiGbc2gaTjabcggaHjabcIha4bWcbaGaem4AaSgabeaakmaabmaabaGafmiEaGNbaebadaWgaaWcbaGaeGimaaJaeiilaWIaem4AaSgabeaaaOGaayjkaiaawMcaaiabgkHiTmaaxababaGagiyBa0MaeiyAaKMaeiOBa4galeaacqWGRbWAaeqaaOWaaeWaaeaacuWG4baEgaqeamaaBaaaleaacqaIWaamcqGGSaalcqWGRbWAaeqaaaGccaGLOaGaayzkaaaaaa@4ECF@

The results are shown in Table 2. We observe that for datasets with large *K *(such as BRN, GCM, and NCI60) the range *R*(x¯0,k
 MathType@MTEF@5@5@+=feaafiart1ev1aaatCvAUfKttLearuWrP9MDH5MBPbIqV92AaeXatLxBI9gBaebbnrfifHhDYfgasaacH8akY=wiFfYdH8Gipec8Eeeu0xXdbba9frFj0=OqFfea0dXdd9vqai=hGuQ8kuc9pgc9s8qqaq=dirpe0xb9q8qiLsFr0=vr0=vr0dc8meaabaqaciaacaGaaeqabaqabeGadaaakeaacuWG4baEgaqeamaaBaaaleaacqaIWaamcqGGSaalcqWGRbWAaeqaaaaa@3196@) is greater than in case of datasets with smaller *K*. Looking at the maximum and minimum values of x¯0,k
 MathType@MTEF@5@5@+=feaafiart1ev1aaatCvAUfKttLearuWrP9MDH5MBPbIqV92AaeXatLxBI9gBaebbnrfifHhDYfgasaacH8akY=wiFfYdH8Gipec8Eeeu0xXdbba9frFj0=OqFfea0dXdd9vqai=hGuQ8kuc9pgc9s8qqaq=dirpe0xb9q8qiLsFr0=vr0=vr0dc8meaabaqaciaacaGaaeqabaqabeGadaaakeaacuWG4baEgaqeamaaBaaaleaacqaIWaamcqGGSaalcqWGRbWAaeqaaaaa@3196@ across *k *in Table 2, we can say with certainty that there is an imbalance among classes in terms of class means especially in datasets containing more than 6 classes. We expect this imbalance to either increase or at least remain unchanged as *K *increases beyond 14 (which is the largest number of classes to be found among real-life datasets). Therefore the implementation of unequal maximum limits to the absolute value of the class means for different classes in equations (4) and (5) is justified, particularly in analyses involving *K *as high as 30 for toy datasets, as is the case in this study.

**Table 2:** Range of class means in real-life datasets.

Dataset	max⁡k(x¯0,k) MathType@MTEF@5@5@+=feaafiart1ev1aaatCvAUfKttLearuWrP9MDH5MBPbIqV92AaeXatLxBI9gBaebbnrfifHhDYfgasaacH8akY=wiFfYdH8Gipec8Eeeu0xXdbba9frFj0=OqFfea0dXdd9vqai=hGuQ8kuc9pgc9s8qqaq=dirpe0xb9q8qiLsFr0=vr0=vr0dc8meaabaqaciaacaGaaeqabaqabeGadaaakeaadaWfqaqaaiGbc2gaTjabcggaHjabcIha4bWcbaGaem4AaSgabeaakmaabmaabaGafmiEaGNbaebadaWgaaWcbaGaeGimaaJaeiilaWIaem4AaSgabeaaaOGaayjkaiaawMcaaaaa@38F1@	min⁡k(x¯0,k) MathType@MTEF@5@5@+=feaafiart1ev1aaatCvAUfKttLearuWrP9MDH5MBPbIqV92AaeXatLxBI9gBaebbnrfifHhDYfgasaacH8akY=wiFfYdH8Gipec8Eeeu0xXdbba9frFj0=OqFfea0dXdd9vqai=hGuQ8kuc9pgc9s8qqaq=dirpe0xb9q8qiLsFr0=vr0=vr0dc8meaabaqaciaacaGaaeqabaqabeGadaaakeaadaWfqaqaaiGbc2gaTjabcMgaPjabc6gaUbWcbaGaem4AaSgabeaakmaabmaabaGafmiEaGNbaebadaWgaaWcbaGaeGimaaJaeiilaWIaem4AaSgabeaaaOGaayjkaiaawMcaaaaa@38ED@	*R*(x¯0,k MathType@MTEF@5@5@+=feaafiart1ev1aaatCvAUfKttLearuWrP9MDH5MBPbIqV92AaeXatLxBI9gBaebbnrfifHhDYfgasaacH8akY=wiFfYdH8Gipec8Eeeu0xXdbba9frFj0=OqFfea0dXdd9vqai=hGuQ8kuc9pgc9s8qqaq=dirpe0xb9q8qiLsFr0=vr0=vr0dc8meaabaqaciaacaGaaeqabaqabeGadaaakeaacuWG4baEgaqeamaaBaaaleaacqaIWaamcqGGSaalcqWGRbWAaeqaaaaa@3196@)
BRN	11.57	3.93	7.65
GCM	73.00	7.77	65.23
NCI60	7.64	4.20	3.44
PDL	2.77	2.67	0.10
Lung	9.62	8.39	1.23
SRBC	2.27	1.59	0.68
MLL	4.35	4.08	0.27
AML/ALL	7.14	6.76	0.38

### Reapplying the analyses on real-life datasets

For real-life datasets, the analyses are implemented separately upon the training set of each split, there being a total of 10 splits of training and test sets. The mean across all splits is taken for the characteristics measured in the analyses: d¯E,α
 MathType@MTEF@5@5@+=feaafiart1ev1aaatCvAUfKttLearuWrP9MDH5MBPbIqV92AaeXatLxBI9gBaebbnrfifHhDYfgasaacH8akY=wiFfYdH8Gipec8Eeeu0xXdbba9frFj0=OqFfea0dXdd9vqai=hGuQ8kuc9pgc9s8qqaq=dirpe0xb9q8qiLsFr0=vr0=vr0dc8meaabaqaciaacaGaaeqabaqabeGadaaakeaacuWGKbazgaqeamaaBaaaleaacqWGfbqrcqGGSaaliiGacqWFXoqyaeqaaaaa@31DA@, d¯MP,α
 MathType@MTEF@5@5@+=feaafiart1ev1aaatCvAUfKttLearuWrP9MDH5MBPbIqV92AaeXatLxBI9gBaebbnrfifHhDYfgasaacH8akY=wiFfYdH8Gipec8Eeeu0xXdbba9frFj0=OqFfea0dXdd9vqai=hGuQ8kuc9pgc9s8qqaq=dirpe0xb9q8qiLsFr0=vr0=vr0dc8meaabaqaciaacaGaaeqabaqabeGadaaakeaacuWGKbazgaqeamaaBaaaleaacqWGnbqtcqWGqbaucqGGSaaliiGacqWFXoqyaeqaaaaa@3313@, and U′Sα
 MathType@MTEF@5@5@+=feaafiart1ev1aaatCvAUfKttLearuWrP9MDH5MBPbIqV92AaeXatLxBI9gBaebbnrfifHhDYfgasaacH8akY=wiFfYdH8Gipec8Eeeu0xXdbba9frFj0=OqFfea0dXdd9vqai=hGuQ8kuc9pgc9s8qqaq=dirpe0xb9q8qiLsFr0=vr0=vr0dc8meaabaqaciaacaGaaeqabaqabeGadaaakeaacuWGvbqvgaqbamaaBaaaleaacqWGtbWudaWgaaadbaacciGae8xSdegabeaaaSqabaaaaa@3124@, and then used to find the corresponding value of the DDP which optimizes each of the aforementioned characteristics.

We will assume that the optimal *P *for each real-life dataset is also directly proportional to *K *(as is the case for toy datasets). However, allowing for remnant noise (left even after data preprocessing), we assign larger values to *P *for real-life datasets (30*K*) than for toy datasets with similar *K*. Furthermore, we conduct two versions of the analyses involving PCA-transformed predictor sets:

• in the first version, only the top three PCs are used

• in the second version, all PCs are used, as is the case for toy datasets.

The reason for this is given as follows: The large percentage of the total variance among samples which is represented by the top PCs is 'relevant' variance (i.e., variance which is due to the difference among classes and thus is relevant w.r.t. the target class concept). On the other hand, the last PCs contain the remainder (small) percentage of the total variance, which is most likely caused by noise or variance within class (i.e., 'non-relevant' variance as opposed to the first type of variance since variance within class is not relevant w.r.t. the target class concept). Variance within class in real-life datasets, unlike variance within class in toy datasets (which is fixed at 1 in this study), differs from class to class even within the same dataset, and is likely to be larger than 1. This is the reason for the first version of the analysis.

**Figure 5:**
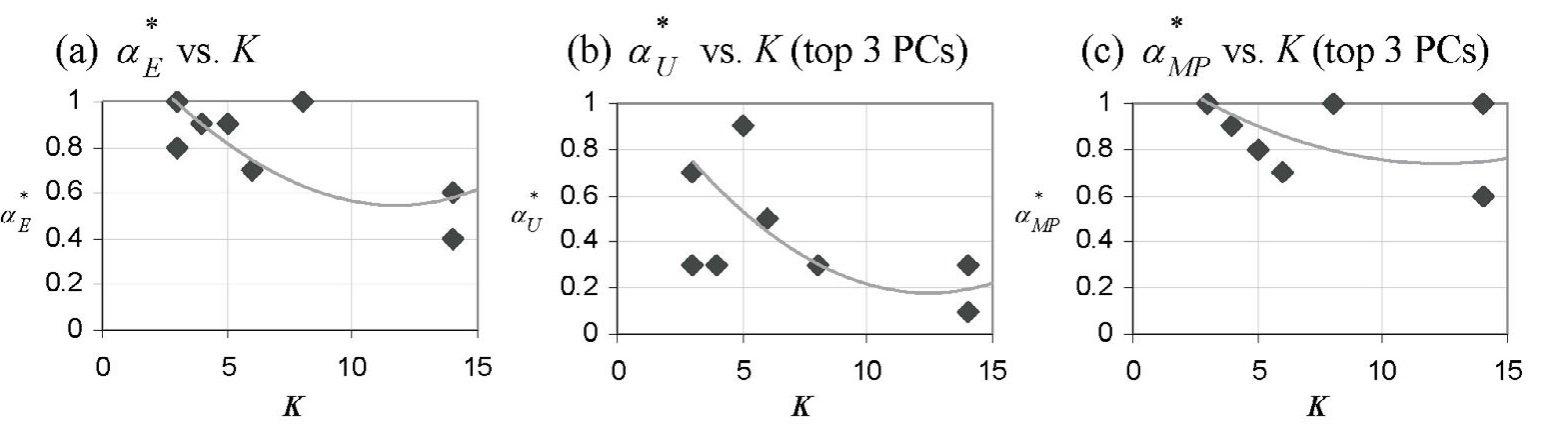
**Analyses on real-life microarray datasets**. Values of the DDP which optimize various predictor set characteristics, (a) αE∗
 MathType@MTEF@5@5@+=feaafiart1ev1aaatCvAUfKttLearuWrP9MDH5MBPbIqV92AaeXatLxBI9gBaebbnrfifHhDYfgasaacH8akY=wiFfYdH8Gipec8Eeeu0xXdbba9frFj0=OqFfea0dXdd9vqai=hGuQ8kuc9pgc9s8qqaq=dirpe0xb9q8qiLsFr0=vr0=vr0dc8meaabaqaciaacaGaaeqabaqabeGadaaakeaaiiGacqWFXoqydaqhaaWcbaGaemyraueabaGaey4fIOcaaaaa@3081@, (b) αU∗
 MathType@MTEF@5@5@+=feaafiart1ev1aaatCvAUfKttLearuWrP9MDH5MBPbIqV92AaeXatLxBI9gBaebbnrfifHhDYfgasaacH8akY=wiFfYdH8Gipec8Eeeu0xXdbba9frFj0=OqFfea0dXdd9vqai=hGuQ8kuc9pgc9s8qqaq=dirpe0xb9q8qiLsFr0=vr0=vr0dc8meaabaqaciaacaGaaeqabaqabeGadaaakeaaiiGacqWFXoqydaqhaaWcbaGaemyvaufabaGaey4fIOcaaaaa@30A1@ (only the top three PCs used), and (c) αMP∗
 MathType@MTEF@5@5@+=feaafiart1ev1aaatCvAUfKttLearuWrP9MDH5MBPbIqV92AaeXatLxBI9gBaebbnrfifHhDYfgasaacH8akY=wiFfYdH8Gipec8Eeeu0xXdbba9frFj0=OqFfea0dXdd9vqai=hGuQ8kuc9pgc9s8qqaq=dirpe0xb9q8qiLsFr0=vr0=vr0dc8meaabaqaciaacaGaaeqabaqabeGadaaakeaaiiGacqWFXoqydaqhaaWcbaGaemyta0KaemiuaafabaGaey4fIOcaaaaa@31BA@ (only the top three PCs used), each as a function of *K *for real-life microarray datasets.

Figure 5 shows that for majority of real-life datasets, the trend regarding the aforementioned three characteristics is similar to the trend for toy datasets (Figures 1, 3, and 4), as indicated by the accompanying gray trend-lines. There are several divergences. In the αE∗
 MathType@MTEF@5@5@+=feaafiart1ev1aaatCvAUfKttLearuWrP9MDH5MBPbIqV92AaeXatLxBI9gBaebbnrfifHhDYfgasaacH8akY=wiFfYdH8Gipec8Eeeu0xXdbba9frFj0=OqFfea0dXdd9vqai=hGuQ8kuc9pgc9s8qqaq=dirpe0xb9q8qiLsFr0=vr0=vr0dc8meaabaqaciaacaGaaeqabaqabeGadaaakeaaiiGacqWFXoqydaqhaaWcbaGaemyraueabaGaey4fIOcaaaaa@3081@ - *K *plot, one dataset (NCI60) produces a point (αE∗
 MathType@MTEF@5@5@+=feaafiart1ev1aaatCvAUfKttLearuWrP9MDH5MBPbIqV92AaeXatLxBI9gBaebbnrfifHhDYfgasaacH8akY=wiFfYdH8Gipec8Eeeu0xXdbba9frFj0=OqFfea0dXdd9vqai=hGuQ8kuc9pgc9s8qqaq=dirpe0xb9q8qiLsFr0=vr0=vr0dc8meaabaqaciaacaGaaeqabaqabeGadaaakeaaiiGacqWFXoqydaqhaaWcbaGaemyraueabaGaey4fIOcaaaaa@3081@ = 1 at *K *= 8) which diverges from the αE∗
 MathType@MTEF@5@5@+=feaafiart1ev1aaatCvAUfKttLearuWrP9MDH5MBPbIqV92AaeXatLxBI9gBaebbnrfifHhDYfgasaacH8akY=wiFfYdH8Gipec8Eeeu0xXdbba9frFj0=OqFfea0dXdd9vqai=hGuQ8kuc9pgc9s8qqaq=dirpe0xb9q8qiLsFr0=vr0=vr0dc8meaabaqaciaacaGaaeqabaqabeGadaaakeaaiiGacqWFXoqydaqhaaWcbaGaemyraueabaGaey4fIOcaaaaa@3081@ - *K *plots observed in toy datasets (Figure 1). Two datasets, BRN and NCI60, give 'outlier' points for the αMP∗
 MathType@MTEF@5@5@+=feaafiart1ev1aaatCvAUfKttLearuWrP9MDH5MBPbIqV92AaeXatLxBI9gBaebbnrfifHhDYfgasaacH8akY=wiFfYdH8Gipec8Eeeu0xXdbba9frFj0=OqFfea0dXdd9vqai=hGuQ8kuc9pgc9s8qqaq=dirpe0xb9q8qiLsFr0=vr0=vr0dc8meaabaqaciaacaGaaeqabaqabeGadaaakeaaiiGacqWFXoqydaqhaaWcbaGaemyta0KaemiuaafabaGaey4fIOcaaaaa@31BA@ - *K *plots. In the αU∗
 MathType@MTEF@5@5@+=feaafiart1ev1aaatCvAUfKttLearuWrP9MDH5MBPbIqV92AaeXatLxBI9gBaebbnrfifHhDYfgasaacH8akY=wiFfYdH8Gipec8Eeeu0xXdbba9frFj0=OqFfea0dXdd9vqai=hGuQ8kuc9pgc9s8qqaq=dirpe0xb9q8qiLsFr0=vr0=vr0dc8meaabaqaciaacaGaaeqabaqabeGadaaakeaaiiGacqWFXoqydaqhaaWcbaGaemyvaufabaGaey4fIOcaaaaa@30A1@ - *K *plot, two datasets, SRBC and MLL, produce points which deviate from the rest. The probable causes of these discrepancies are presented in the Discussion section.

**Figure 6:**
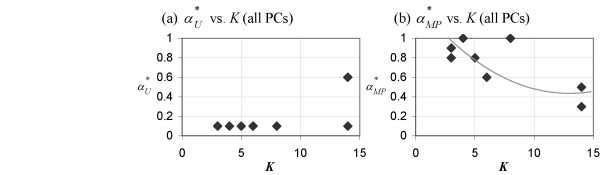
**Analyses on real-life microarray datasets (with all PCs)**. Values of the DDP which optimize various predictor set characteristics, (a) αU∗
 MathType@MTEF@5@5@+=feaafiart1ev1aaatCvAUfKttLearuWrP9MDH5MBPbIqV92AaeXatLxBI9gBaebbnrfifHhDYfgasaacH8akY=wiFfYdH8Gipec8Eeeu0xXdbba9frFj0=OqFfea0dXdd9vqai=hGuQ8kuc9pgc9s8qqaq=dirpe0xb9q8qiLsFr0=vr0=vr0dc8meaabaqaciaacaGaaeqabaqabeGadaaakeaaiiGacqWFXoqydaqhaaWcbaGaemyvaufabaGaey4fIOcaaaaa@30A1@, and (b) αMP∗
 MathType@MTEF@5@5@+=feaafiart1ev1aaatCvAUfKttLearuWrP9MDH5MBPbIqV92AaeXatLxBI9gBaebbnrfifHhDYfgasaacH8akY=wiFfYdH8Gipec8Eeeu0xXdbba9frFj0=OqFfea0dXdd9vqai=hGuQ8kuc9pgc9s8qqaq=dirpe0xb9q8qiLsFr0=vr0=vr0dc8meaabaqaciaacaGaaeqabaqabeGadaaakeaaiiGacqWFXoqydaqhaaWcbaGaemyta0KaemiuaafabaGaey4fIOcaaaaa@31BA@ (both with all PCs used), each as a function of *K *for real-life microarray datasets.

In Figure 6, we repeat the analysis involving PCA using all PCs instead of merely the top three PCs. This applies only to the αMP∗
 MathType@MTEF@5@5@+=feaafiart1ev1aaatCvAUfKttLearuWrP9MDH5MBPbIqV92AaeXatLxBI9gBaebbnrfifHhDYfgasaacH8akY=wiFfYdH8Gipec8Eeeu0xXdbba9frFj0=OqFfea0dXdd9vqai=hGuQ8kuc9pgc9s8qqaq=dirpe0xb9q8qiLsFr0=vr0=vr0dc8meaabaqaciaacaGaaeqabaqabeGadaaakeaaiiGacqWFXoqydaqhaaWcbaGaemyta0KaemiuaafabaGaey4fIOcaaaaa@31BA@ - *K *and αU∗
 MathType@MTEF@5@5@+=feaafiart1ev1aaatCvAUfKttLearuWrP9MDH5MBPbIqV92AaeXatLxBI9gBaebbnrfifHhDYfgasaacH8akY=wiFfYdH8Gipec8Eeeu0xXdbba9frFj0=OqFfea0dXdd9vqai=hGuQ8kuc9pgc9s8qqaq=dirpe0xb9q8qiLsFr0=vr0=vr0dc8meaabaqaciaacaGaaeqabaqabeGadaaakeaaiiGacqWFXoqydaqhaaWcbaGaemyvaufabaGaey4fIOcaaaaa@30A1@ - *K *plots, since the computation of αE∗
 MathType@MTEF@5@5@+=feaafiart1ev1aaatCvAUfKttLearuWrP9MDH5MBPbIqV92AaeXatLxBI9gBaebbnrfifHhDYfgasaacH8akY=wiFfYdH8Gipec8Eeeu0xXdbba9frFj0=OqFfea0dXdd9vqai=hGuQ8kuc9pgc9s8qqaq=dirpe0xb9q8qiLsFr0=vr0=vr0dc8meaabaqaciaacaGaaeqabaqabeGadaaakeaaiiGacqWFXoqydaqhaaWcbaGaemyraueabaGaey4fIOcaaaaa@3081@ does not involve PCA. We find that the αMP∗
 MathType@MTEF@5@5@+=feaafiart1ev1aaatCvAUfKttLearuWrP9MDH5MBPbIqV92AaeXatLxBI9gBaebbnrfifHhDYfgasaacH8akY=wiFfYdH8Gipec8Eeeu0xXdbba9frFj0=OqFfea0dXdd9vqai=hGuQ8kuc9pgc9s8qqaq=dirpe0xb9q8qiLsFr0=vr0=vr0dc8meaabaqaciaacaGaaeqabaqabeGadaaakeaaiiGacqWFXoqydaqhaaWcbaGaemyta0KaemiuaafabaGaey4fIOcaaaaa@31BA@ - *K *plot for real-life datasets obtained by using all PCs still resembles the αMP∗
 MathType@MTEF@5@5@+=feaafiart1ev1aaatCvAUfKttLearuWrP9MDH5MBPbIqV92AaeXatLxBI9gBaebbnrfifHhDYfgasaacH8akY=wiFfYdH8Gipec8Eeeu0xXdbba9frFj0=OqFfea0dXdd9vqai=hGuQ8kuc9pgc9s8qqaq=dirpe0xb9q8qiLsFr0=vr0=vr0dc8meaabaqaciaacaGaaeqabaqabeGadaaakeaaiiGacqWFXoqydaqhaaWcbaGaemyta0KaemiuaafabaGaey4fIOcaaaaa@31BA@ - *K *plots from toy datasets (Figure 4). Conversely, the αU∗
 MathType@MTEF@5@5@+=feaafiart1ev1aaatCvAUfKttLearuWrP9MDH5MBPbIqV92AaeXatLxBI9gBaebbnrfifHhDYfgasaacH8akY=wiFfYdH8Gipec8Eeeu0xXdbba9frFj0=OqFfea0dXdd9vqai=hGuQ8kuc9pgc9s8qqaq=dirpe0xb9q8qiLsFr0=vr0=vr0dc8meaabaqaciaacaGaaeqabaqabeGadaaakeaaiiGacqWFXoqydaqhaaWcbaGaemyvaufabaGaey4fIOcaaaaa@30A1@ - *K *plot for real-life datasets obtained by using all PCs shows no resemblance whatsoever to the αU∗
 MathType@MTEF@5@5@+=feaafiart1ev1aaatCvAUfKttLearuWrP9MDH5MBPbIqV92AaeXatLxBI9gBaebbnrfifHhDYfgasaacH8akY=wiFfYdH8Gipec8Eeeu0xXdbba9frFj0=OqFfea0dXdd9vqai=hGuQ8kuc9pgc9s8qqaq=dirpe0xb9q8qiLsFr0=vr0=vr0dc8meaabaqaciaacaGaaeqabaqabeGadaaakeaaiiGacqWFXoqydaqhaaWcbaGaemyvaufabaGaey4fIOcaaaaa@30A1@ - *K *plots from toy datasets (Figure 3). We deduce that αU∗
 MathType@MTEF@5@5@+=feaafiart1ev1aaatCvAUfKttLearuWrP9MDH5MBPbIqV92AaeXatLxBI9gBaebbnrfifHhDYfgasaacH8akY=wiFfYdH8Gipec8Eeeu0xXdbba9frFj0=OqFfea0dXdd9vqai=hGuQ8kuc9pgc9s8qqaq=dirpe0xb9q8qiLsFr0=vr0=vr0dc8meaabaqaciaacaGaaeqabaqabeGadaaakeaaiiGacqWFXoqydaqhaaWcbaGaemyvaufabaGaey4fIOcaaaaa@30A1@ is more sensitive to the addition of the within-class variance and noise introduced by the inclusion of the rest of the PCs (i.e., the fourth and the subsequent PCs) than αMP∗
 MathType@MTEF@5@5@+=feaafiart1ev1aaatCvAUfKttLearuWrP9MDH5MBPbIqV92AaeXatLxBI9gBaebbnrfifHhDYfgasaacH8akY=wiFfYdH8Gipec8Eeeu0xXdbba9frFj0=OqFfea0dXdd9vqai=hGuQ8kuc9pgc9s8qqaq=dirpe0xb9q8qiLsFr0=vr0=vr0dc8meaabaqaciaacaGaaeqabaqabeGadaaakeaaiiGacqWFXoqydaqhaaWcbaGaemyta0KaemiuaafabaGaey4fIOcaaaaa@31BA@ is.

## Discussion

In this section, we demonstrate how the DDP works for datasets with different number of classes. Then we discuss the reasons for the difference between the behaviors of αE∗
 MathType@MTEF@5@5@+=feaafiart1ev1aaatCvAUfKttLearuWrP9MDH5MBPbIqV92AaeXatLxBI9gBaebbnrfifHhDYfgasaacH8akY=wiFfYdH8Gipec8Eeeu0xXdbba9frFj0=OqFfea0dXdd9vqai=hGuQ8kuc9pgc9s8qqaq=dirpe0xb9q8qiLsFr0=vr0=vr0dc8meaabaqaciaacaGaaeqabaqabeGadaaakeaaiiGacqWFXoqydaqhaaWcbaGaemyraueabaGaey4fIOcaaaaa@3081@ and αM∗
 MathType@MTEF@5@5@+=feaafiart1ev1aaatCvAUfKttLearuWrP9MDH5MBPbIqV92AaeXatLxBI9gBaebbnrfifHhDYfgasaacH8akY=wiFfYdH8Gipec8Eeeu0xXdbba9frFj0=OqFfea0dXdd9vqai=hGuQ8kuc9pgc9s8qqaq=dirpe0xb9q8qiLsFr0=vr0=vr0dc8meaabaqaciaacaGaaeqabaqabeGadaaakeaaiiGacqWFXoqydaqhaaWcbaGaemyta0eabaGaey4fIOcaaaaa@3091@ as *K *increases and the causes of the discrepancies between the plots for toy datasets and real-life datasets.

### A look at how the DDP concept works

**Figure 7:**
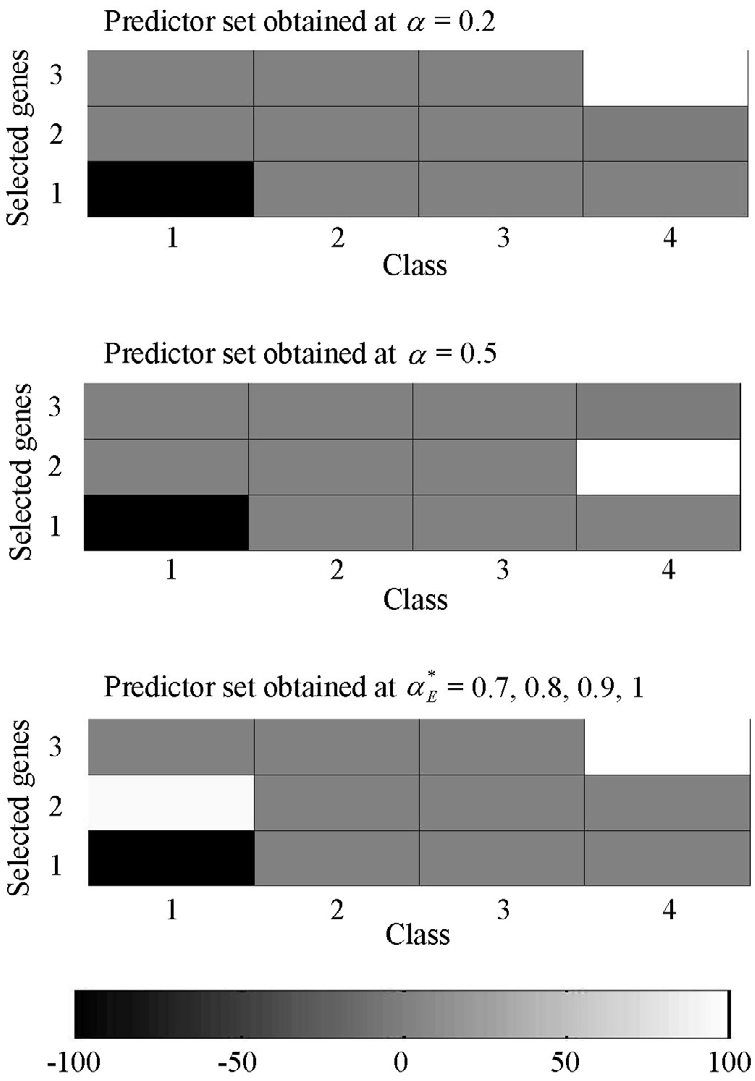
**Separation of classes for a 4-class OVA-based toy dataset**. Separation of classes by predictor sets obtained using several values of the DDP: 0.2, 0.5 (equal-priorities scoring method), and αE∗
 MathType@MTEF@5@5@+=feaafiart1ev1aaatCvAUfKttLearuWrP9MDH5MBPbIqV92AaeXatLxBI9gBaebbnrfifHhDYfgasaacH8akY=wiFfYdH8Gipec8Eeeu0xXdbba9frFj0=OqFfea0dXdd9vqai=hGuQ8kuc9pgc9s8qqaq=dirpe0xb9q8qiLsFr0=vr0=vr0dc8meaabaqaciaacaGaaeqabaqabeGadaaakeaaiiGacqWFXoqydaqhaaWcbaGaemyraueabaGaey4fIOcaaaaa@3081@ for a 4-class OVA-based toy dataset. The mean of the *i*-th member of a predictor set across samples belonging to class *k *is represented by the intensity of the grayscale shading in a rectangular patch located on the *i*-th row and the *k*-th column.

**Figure 8:**
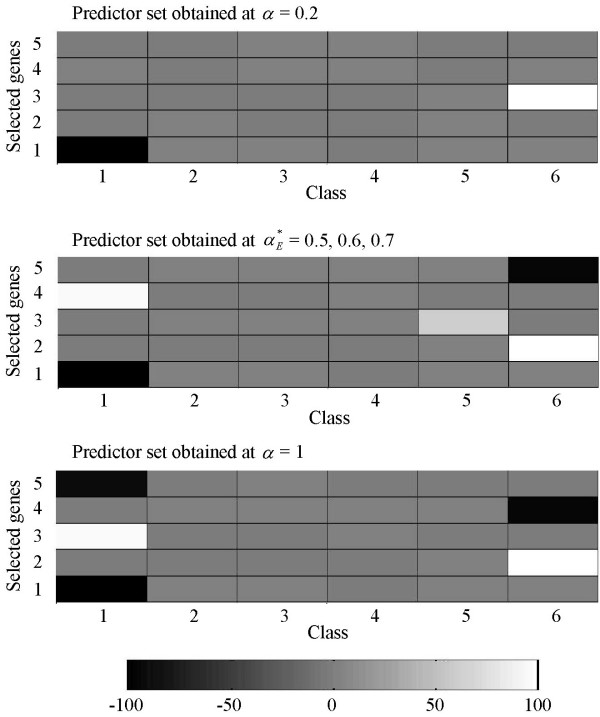
**Separation of classes for a 6-class OVA-based toy dataset**. Separation of classes by predictor sets obtained using several values of the DDP: 0.2, αE∗
 MathType@MTEF@5@5@+=feaafiart1ev1aaatCvAUfKttLearuWrP9MDH5MBPbIqV92AaeXatLxBI9gBaebbnrfifHhDYfgasaacH8akY=wiFfYdH8Gipec8Eeeu0xXdbba9frFj0=OqFfea0dXdd9vqai=hGuQ8kuc9pgc9s8qqaq=dirpe0xb9q8qiLsFr0=vr0=vr0dc8meaabaqaciaacaGaaeqabaqabeGadaaakeaaiiGacqWFXoqydaqhaaWcbaGaemyraueabaGaey4fIOcaaaaa@3081@, and 1 (rank-based selection) for a 6-class OVA-based toy dataset. The mean of the *i*-th member of a predictor set across samples belonging to class *k *is represented by the intensity of the grayscale shading in a rectangular patch located on the *i*-th row and the *k*-th column.

**Figure 9:**
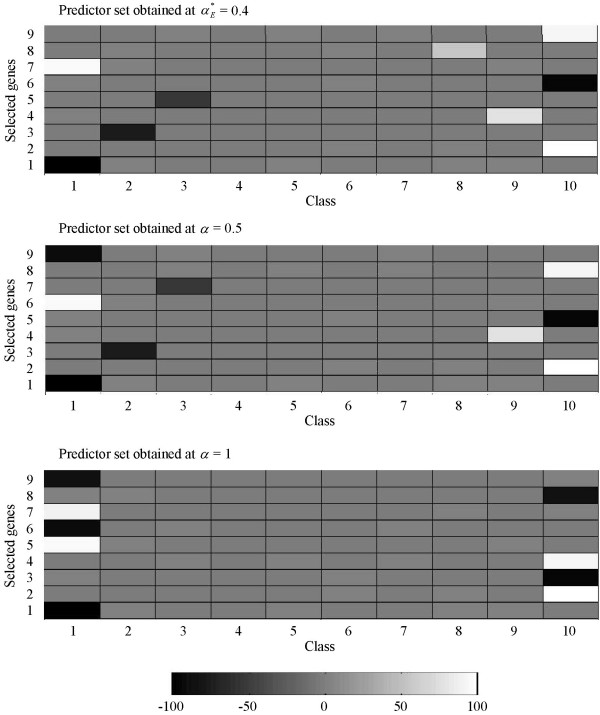
**Separation of classes for a 10-class OVA-based toy dataset**. Separation of classes by predictor sets obtained using several values of the DDP: αE∗
 MathType@MTEF@5@5@+=feaafiart1ev1aaatCvAUfKttLearuWrP9MDH5MBPbIqV92AaeXatLxBI9gBaebbnrfifHhDYfgasaacH8akY=wiFfYdH8Gipec8Eeeu0xXdbba9frFj0=OqFfea0dXdd9vqai=hGuQ8kuc9pgc9s8qqaq=dirpe0xb9q8qiLsFr0=vr0=vr0dc8meaabaqaciaacaGaaeqabaqabeGadaaakeaaiiGacqWFXoqydaqhaaWcbaGaemyraueabaGaey4fIOcaaaaa@3081@, 0.5 (equal-priorities scoring method), and 1 (rank-based selection) for a 10-class OVA-based toy dataset. The mean of the *i*-th member of a predictor set across samples belonging to class *k *is represented by the intensity of the grayscale shading in a rectangular patch located on the *i*-th row and the *k*-th column.

**Figure 10:**
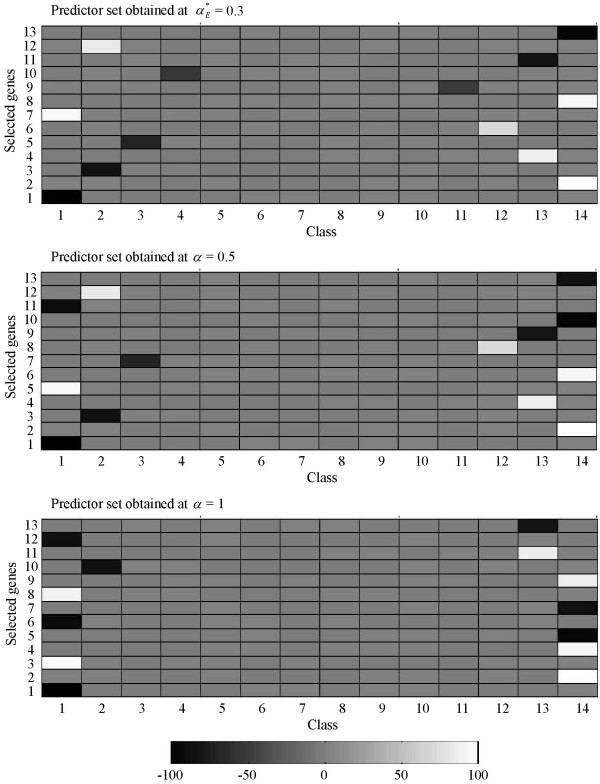
**Separation of classes for a 14-class OVA-based toy dataset**. Separation of classes by predictor sets obtained using several values of the DDP: αE∗
 MathType@MTEF@5@5@+=feaafiart1ev1aaatCvAUfKttLearuWrP9MDH5MBPbIqV92AaeXatLxBI9gBaebbnrfifHhDYfgasaacH8akY=wiFfYdH8Gipec8Eeeu0xXdbba9frFj0=OqFfea0dXdd9vqai=hGuQ8kuc9pgc9s8qqaq=dirpe0xb9q8qiLsFr0=vr0=vr0dc8meaabaqaciaacaGaaeqabaqabeGadaaakeaaiiGacqWFXoqydaqhaaWcbaGaemyraueabaGaey4fIOcaaaaa@3081@, 0.5 (equal-priorities scoring method), and 1 (rank-based selection) for a 14-class OVA-based toy dataset. The mean of the *i*-th member of a predictor set across samples belonging to class *k *is represented by the intensity of the grayscale shading in a rectangular patch located on the *i*-th row and the *k*-th column.

A few examples from OVA-based toy datasets generated at *G *= 30 are used to demonstrate how the DDP concept works for different number of classes. Figures 7, 8, 9, 10 show predictor sets obtained using several values of the DDP: αE∗
 MathType@MTEF@5@5@+=feaafiart1ev1aaatCvAUfKttLearuWrP9MDH5MBPbIqV92AaeXatLxBI9gBaebbnrfifHhDYfgasaacH8akY=wiFfYdH8Gipec8Eeeu0xXdbba9frFj0=OqFfea0dXdd9vqai=hGuQ8kuc9pgc9s8qqaq=dirpe0xb9q8qiLsFr0=vr0=vr0dc8meaabaqaciaacaGaaeqabaqabeGadaaakeaaiiGacqWFXoqydaqhaaWcbaGaemyraueabaGaey4fIOcaaaaa@3081@, 0.5 (equal-priorities scoring method), and 1 (rank-based selection) for datasets with *K *= 4, 6, 10, and 14 classes respectively. (See the Background section for details on the significance of the values of the DDP.) The intensity of the grayscale shading in a rectangular patch located on the *i*-th row and the *k*-th column indicates the value of the mean of the *i*-th member of the predictor set across samples belonging to class *k*.

For the 4-class toy dataset, the values of the DDP which maximize separation of classes in terms of the Euclidean distance are 0.7, 0.8, 0.9, and 1; we can observe from Figure 7 that the predictor set produced from these DDP values do not contain non-relevant genes (genes which are generated at large values of *g *and hence barely differentiate among the *K *classes). On the other hand, predictor sets obtained using the DDP values of 0.5 and smaller *do *comprise such non-relevant genes.

Figure 8 shows that for the 6-class toy dataset, only predictor sets obtained at the DDP values of αE∗
 MathType@MTEF@5@5@+=feaafiart1ev1aaatCvAUfKttLearuWrP9MDH5MBPbIqV92AaeXatLxBI9gBaebbnrfifHhDYfgasaacH8akY=wiFfYdH8Gipec8Eeeu0xXdbba9frFj0=OqFfea0dXdd9vqai=hGuQ8kuc9pgc9s8qqaq=dirpe0xb9q8qiLsFr0=vr0=vr0dc8meaabaqaciaacaGaaeqabaqabeGadaaakeaaiiGacqWFXoqydaqhaaWcbaGaemyraueabaGaey4fIOcaaaaa@3081@ (0.5, 0.6, or 0.7) are able to differentiate samples among the maximum number of classes (3 classes: class 1, class 5, and class 6). Predictor sets obtained at *α *= 1 and DDP values less than 0.5 (e.g., *α *= 0.2) merely manage to distinguish samples between 2 classes at most (class 1 and class 6). The predictor set obtained at *α *= 1 contains redundant marker genes from both the first and sixth groups of marker genes. The predictor set obtained at *α *= 0.2 contains no such redundancy but has non-relevant genes among its members.

Note that at smaller values of *K*, αE∗
 MathType@MTEF@5@5@+=feaafiart1ev1aaatCvAUfKttLearuWrP9MDH5MBPbIqV92AaeXatLxBI9gBaebbnrfifHhDYfgasaacH8akY=wiFfYdH8Gipec8Eeeu0xXdbba9frFj0=OqFfea0dXdd9vqai=hGuQ8kuc9pgc9s8qqaq=dirpe0xb9q8qiLsFr0=vr0=vr0dc8meaabaqaciaacaGaaeqabaqabeGadaaakeaaiiGacqWFXoqydaqhaaWcbaGaemyraueabaGaey4fIOcaaaaa@3081@ tends to have multiple values; this is because more than one value of *α *satisfies the requirement (d¯E,α)
 MathType@MTEF@5@5@+=feaafiart1ev1aaatCvAUfKttLearuWrP9MDH5MBPbIqV92AaeXatLxBI9gBaebbnrfifHhDYfgasaacH8akY=wiFfYdH8Gipec8Eeeu0xXdbba9frFj0=OqFfea0dXdd9vqai=hGuQ8kuc9pgc9s8qqaq=dirpe0xb9q8qiLsFr0=vr0=vr0dc8meaabaqaciaacaGaaeqabaqabeGadaaakeaadaqadaqaaiqbdsgaKzaaraWaaSbaaSqaaiabdweafjabcYcaSGGaciab=f7aHbqabaaakiaawIcacaGLPaaaaaa@336D@ for datasets where *K *< 9.

For the 10-class toy dataset, the predictor set obtained at αE∗
 MathType@MTEF@5@5@+=feaafiart1ev1aaatCvAUfKttLearuWrP9MDH5MBPbIqV92AaeXatLxBI9gBaebbnrfifHhDYfgasaacH8akY=wiFfYdH8Gipec8Eeeu0xXdbba9frFj0=OqFfea0dXdd9vqai=hGuQ8kuc9pgc9s8qqaq=dirpe0xb9q8qiLsFr0=vr0=vr0dc8meaabaqaciaacaGaaeqabaqabeGadaaakeaaiiGacqWFXoqydaqhaaWcbaGaemyraueabaGaey4fIOcaaaaa@3081@ = 0.4 contains representatives from the largest number of groups of marker genes, 6 (Figure 9). This means that this predictor set is able to distinguish samples among those 6 classes (class 1, class 2, class 3, class 8, class 9, and class 10). The predictor set found from the equal-priorities scoring method (*α *= 0.5) is only capable of telling apart samples from 5 classes and contains more redundancy than the optimal predictor set. For instance, *S*_0.5 _has two redundant marker genes from the first group of marker genes, whereas *S*_0.4 _has only one redundant marker gene from that same group of marker genes. The rank-based predictor set (*α *= 1) comprises redundant marker genes from only 2 groups of marker genes and therefore can only differentiate samples of the associated 2 classes from any other classes.

Figure 10 shows that for the 14-class toy dataset, the predictor set obtained at αE∗
 MathType@MTEF@5@5@+=feaafiart1ev1aaatCvAUfKttLearuWrP9MDH5MBPbIqV92AaeXatLxBI9gBaebbnrfifHhDYfgasaacH8akY=wiFfYdH8Gipec8Eeeu0xXdbba9frFj0=OqFfea0dXdd9vqai=hGuQ8kuc9pgc9s8qqaq=dirpe0xb9q8qiLsFr0=vr0=vr0dc8meaabaqaciaacaGaaeqabaqabeGadaaakeaaiiGacqWFXoqydaqhaaWcbaGaemyraueabaGaey4fIOcaaaaa@3081@ = 0.3, *S*_0.3 _contains genes from 8 groups of marker genes and thus is able to distinguish samples among *more *classes than predictor sets obtained using any other values of the DDP. The equal-priorities scoring method produces the predictor set *S*_0.5 _which is not able to differentiate samples of class 4 and class 11 from all other classes because the members of *S*_0.5 _come from only 6 groups of marker genes. *S*_0.5 _also contains more redundancy than *S*_0.3_. *S*_0.5 _has 2 and 3 redundant genes from the first and 14-th groups of marker genes, respectively, while *S*_0.3 _has only 1 and 2 redundant genes from the first and 14-th groups of marker genes, respectively. The rank-based predictor set *S*_1 _fares worse since it can only tell apart samples from 4 classes due to the high redundancy among its members (i.e., redundant genes from 3 out of the 4 groups of marker genes).

In summary, a predictor set obtained at αE∗
 MathType@MTEF@5@5@+=feaafiart1ev1aaatCvAUfKttLearuWrP9MDH5MBPbIqV92AaeXatLxBI9gBaebbnrfifHhDYfgasaacH8akY=wiFfYdH8Gipec8Eeeu0xXdbba9frFj0=OqFfea0dXdd9vqai=hGuQ8kuc9pgc9s8qqaq=dirpe0xb9q8qiLsFr0=vr0=vr0dc8meaabaqaciaacaGaaeqabaqabeGadaaakeaaiiGacqWFXoqydaqhaaWcbaGaemyraueabaGaey4fIOcaaaaa@3081@ contains representative genes from *more *groups of marker genes and thus has lower redundancy compared to predictor sets found using any other values of the DDP. As mentioned previously, the value of αE∗
 MathType@MTEF@5@5@+=feaafiart1ev1aaatCvAUfKttLearuWrP9MDH5MBPbIqV92AaeXatLxBI9gBaebbnrfifHhDYfgasaacH8akY=wiFfYdH8Gipec8Eeeu0xXdbba9frFj0=OqFfea0dXdd9vqai=hGuQ8kuc9pgc9s8qqaq=dirpe0xb9q8qiLsFr0=vr0=vr0dc8meaabaqaciaacaGaaeqabaqabeGadaaakeaaiiGacqWFXoqydaqhaaWcbaGaemyraueabaGaey4fIOcaaaaa@3081@ changes depending on the value of *K*. Therefore the equal-priorities scoring method is only capable of producing the optimal predictor set for a certain range of *K*. Below that range (small number of classes), rank-based selection may work better than equal-priorities scoring method (supporting the implication from the 2-class example of [3]), while DDP values smaller than or equal to 0.5 will select non-relevant genes. Above that range (large number of classes), the value of the DDP producing the optimal predictor set is always less than 0.5; *S*_0.5 _will contain more redundancy and, for a given *P*, is able to tell apart samples from *smaller *number of classes than the predictor set found using αE∗
 MathType@MTEF@5@5@+=feaafiart1ev1aaatCvAUfKttLearuWrP9MDH5MBPbIqV92AaeXatLxBI9gBaebbnrfifHhDYfgasaacH8akY=wiFfYdH8Gipec8Eeeu0xXdbba9frFj0=OqFfea0dXdd9vqai=hGuQ8kuc9pgc9s8qqaq=dirpe0xb9q8qiLsFr0=vr0=vr0dc8meaabaqaciaacaGaaeqabaqabeGadaaakeaaiiGacqWFXoqydaqhaaWcbaGaemyraueabaGaey4fIOcaaaaa@3081@.

At *P *equal to *K *- 1 for OVA-based toy datasets we observe that none of the DDP values (whether αE∗
 MathType@MTEF@5@5@+=feaafiart1ev1aaatCvAUfKttLearuWrP9MDH5MBPbIqV92AaeXatLxBI9gBaebbnrfifHhDYfgasaacH8akY=wiFfYdH8Gipec8Eeeu0xXdbba9frFj0=OqFfea0dXdd9vqai=hGuQ8kuc9pgc9s8qqaq=dirpe0xb9q8qiLsFr0=vr0=vr0dc8meaabaqaciaacaGaaeqabaqabeGadaaakeaaiiGacqWFXoqydaqhaaWcbaGaemyraueabaGaey4fIOcaaaaa@3081@, 0.5, or 1) are able to produce predictor sets which contain representatives from at least *K *- 1 different groups of marker genes. This is definitely achievable with greater *P*; we also do not expect the findings in this study to change significantly if a greater value of *P *is used. The predictor set found at αE∗
 MathType@MTEF@5@5@+=feaafiart1ev1aaatCvAUfKttLearuWrP9MDH5MBPbIqV92AaeXatLxBI9gBaebbnrfifHhDYfgasaacH8akY=wiFfYdH8Gipec8Eeeu0xXdbba9frFj0=OqFfea0dXdd9vqai=hGuQ8kuc9pgc9s8qqaq=dirpe0xb9q8qiLsFr0=vr0=vr0dc8meaabaqaciaacaGaaeqabaqabeGadaaakeaaiiGacqWFXoqydaqhaaWcbaGaemyraueabaGaey4fIOcaaaaa@3081@ will always contain representatives from *more *different groups of marker genes than predictor sets obtained using any other values of the DDP, and the value of αE∗
 MathType@MTEF@5@5@+=feaafiart1ev1aaatCvAUfKttLearuWrP9MDH5MBPbIqV92AaeXatLxBI9gBaebbnrfifHhDYfgasaacH8akY=wiFfYdH8Gipec8Eeeu0xXdbba9frFj0=OqFfea0dXdd9vqai=hGuQ8kuc9pgc9s8qqaq=dirpe0xb9q8qiLsFr0=vr0=vr0dc8meaabaqaciaacaGaaeqabaqabeGadaaakeaaiiGacqWFXoqydaqhaaWcbaGaemyraueabaGaey4fIOcaaaaa@3081@ is not necessarily 0.5 (equal-priorities scoring method) or 1 (rank-based selection), but varies according to *K*. Similar findings are also observed for toy datasets generated from the PW model, but for the sake of brevity, are not included in this paper.

### The difference between the behaviors of αE∗
 MathType@MTEF@5@5@+=feaafiart1ev1aaatCvAUfKttLearuWrP9MDH5MBPbIqV92AaeXatLxBI9gBaebbnrfifHhDYfgasaacH8akY=wiFfYdH8Gipec8Eeeu0xXdbba9frFj0=OqFfea0dXdd9vqai=hGuQ8kuc9pgc9s8qqaq=dirpe0xb9q8qiLsFr0=vr0=vr0dc8meaabaqaciaacaGaaeqabaqabeGadaaakeaaiiGacqWFXoqydaqhaaWcbaGaemyraueabaGaey4fIOcaaaaa@3081@ and αM∗
 MathType@MTEF@5@5@+=feaafiart1ev1aaatCvAUfKttLearuWrP9MDH5MBPbIqV92AaeXatLxBI9gBaebbnrfifHhDYfgasaacH8akY=wiFfYdH8Gipec8Eeeu0xXdbba9frFj0=OqFfea0dXdd9vqai=hGuQ8kuc9pgc9s8qqaq=dirpe0xb9q8qiLsFr0=vr0=vr0dc8meaabaqaciaacaGaaeqabaqabeGadaaakeaaiiGacqWFXoqydaqhaaWcbaGaemyta0eabaGaey4fIOcaaaaa@3091@ as *K *increases

The difference that we observe between the αE∗
 MathType@MTEF@5@5@+=feaafiart1ev1aaatCvAUfKttLearuWrP9MDH5MBPbIqV92AaeXatLxBI9gBaebbnrfifHhDYfgasaacH8akY=wiFfYdH8Gipec8Eeeu0xXdbba9frFj0=OqFfea0dXdd9vqai=hGuQ8kuc9pgc9s8qqaq=dirpe0xb9q8qiLsFr0=vr0=vr0dc8meaabaqaciaacaGaaeqabaqabeGadaaakeaaiiGacqWFXoqydaqhaaWcbaGaemyraueabaGaey4fIOcaaaaa@3081@ - *K *plots (Figure 1) and the αM∗
 MathType@MTEF@5@5@+=feaafiart1ev1aaatCvAUfKttLearuWrP9MDH5MBPbIqV92AaeXatLxBI9gBaebbnrfifHhDYfgasaacH8akY=wiFfYdH8Gipec8Eeeu0xXdbba9frFj0=OqFfea0dXdd9vqai=hGuQ8kuc9pgc9s8qqaq=dirpe0xb9q8qiLsFr0=vr0=vr0dc8meaabaqaciaacaGaaeqabaqabeGadaaakeaaiiGacqWFXoqydaqhaaWcbaGaemyta0eabaGaey4fIOcaaaaa@3091@ - *K *plots (Figure 2) is rooted in the fundamental difference between the Euclidean and the Manhattan distances. The Euclidean distance is a square root of the sum of the squared differences between class means, as indicated in equation (7). In contrast, the Manhattan distance is simply the sum of the absolute differences between class means, as indicated in equation (10). Because of this, if there are *h *features that are redundant w.r.t. each other in the predictor set, all *h *features contribute

• the square root of their corresponding differences between class means to the Euclidean distance

• simply the absolute value of their corresponding differences between class means to the Manhattan distance.

If for the sake of simplicity we assume that all *h *features contain the same corresponding differences between class means, Δ*d*, then the Euclidean distance is Δ*d*·h
 MathType@MTEF@5@5@+=feaafiart1ev1aaatCvAUfKttLearuWrP9MDH5MBPbIqV92AaeXatLxBI9gBaebbnrfifHhDYfgasaacH8akY=wiFfYdH8Gipec8Eeeu0xXdbba9frFj0=OqFfea0dXdd9vqai=hGuQ8kuc9pgc9s8qqaq=dirpe0xb9q8qiLsFr0=vr0=vr0dc8meaabaqaciaacaGaaeqabaqabeGadaaakeaadaGcaaqaaiabdIgaObWcbeaaaaa@2E20@ and the Manhattan distance is Δ*d*·*h*. Recall that the higher the value of *h*, the higher the redundancy in the predictor set (and the lower the antiredundancy). Note that redundant features are not necessarily non-relevant. Furthermore, we assume that the other (*P *- *h*) members of the predictor set are equally relevant (i.e., contain the same corresponding differences between class means, Δ*d*).

Since the Manhattan distance in the original feature space gives equal weight to the contributions from the bulk of redundant features (represented by *h*) and from the individual relevance (represented by Δ*d*), it is maximized when relevance is maximized (Δ*d *is maximized) and/or antiredundancy is *minimized *(*h *is maximized), regardless of *K*. In the context of the DDP, there is greater emphasis on maximizing relevance than on maximizing antiredundancy in the range [0.8,1], where majority of the points in the αM∗
 MathType@MTEF@5@5@+=feaafiart1ev1aaatCvAUfKttLearuWrP9MDH5MBPbIqV92AaeXatLxBI9gBaebbnrfifHhDYfgasaacH8akY=wiFfYdH8Gipec8Eeeu0xXdbba9frFj0=OqFfea0dXdd9vqai=hGuQ8kuc9pgc9s8qqaq=dirpe0xb9q8qiLsFr0=vr0=vr0dc8meaabaqaciaacaGaaeqabaqabeGadaaakeaaiiGacqWFXoqydaqhaaWcbaGaemyta0eabaGaey4fIOcaaaaa@3091@ - *K *plots are located (Figure 2). We can then deduce that the Manhattan distance is a pure embodiment of relevance because it does not matter whether the total relevance results from redundancy or otherwise – any relevant feature, whether redundant w.r.t. the other members of the predictor set or not, will increase separation of classes as measured using the Manhattan distance.

On the other hand, the Euclidean distance gives the individual relevance (represented by Δ*d*) more weight than it gives to the number of redundant features, *h*, since it only takes account of the square root of *h *and not *h *by itself as the Manhattan distance does. We denote the separation between a pair of classes as measured using the Euclidean distance as *d*_*e*_, and the separation between a pair of classes as measured using the Manhattan distance as *d*_*m*_. Recalling that *d*_*e *_= Δ*d*·h
 MathType@MTEF@5@5@+=feaafiart1ev1aaatCvAUfKttLearuWrP9MDH5MBPbIqV92AaeXatLxBI9gBaebbnrfifHhDYfgasaacH8akY=wiFfYdH8Gipec8Eeeu0xXdbba9frFj0=OqFfea0dXdd9vqai=hGuQ8kuc9pgc9s8qqaq=dirpe0xb9q8qiLsFr0=vr0=vr0dc8meaabaqaciaacaGaaeqabaqabeGadaaakeaadaGcaaqaaiabdIgaObWcbeaaaaa@2E20@ and *d*_*m *_= Δ*d*·*h*, we note that

de=dmh
 MathType@MTEF@5@5@+=feaafiart1ev1aaatCvAUfKttLearuWrP9MDH5MBPbIqV92AaeXatLxBI9gBaebbnrfifHhDYfgasaacH8akY=wiFfYdH8Gipec8Eeeu0xXdbba9frFj0=OqFfea0dXdd9vqai=hGuQ8kuc9pgc9s8qqaq=dirpe0xb9q8qiLsFr0=vr0=vr0dc8meaabaqaciaacaGaaeqabaqabeGadaaakeaacqWGKbazdaWgaaWcbaGaemyzaugabeaakiabg2da9maalaaabaGaemizaq2aaSbaaSqaaiabd2gaTbqabaaakeaadaGcaaqaaiabdIgaObWcbeaaaaaaaa@34FA@

Given that the Manhattan distance, *d*_*m*_, represents relevance and *h *represents redundancy, then in maximizing separation of classes as measured using the Euclidean distance, *d*_*e*_, we are maximizing relevance while at the same time maximizing antiredundancy. This is the reason that, unlike αM∗
 MathType@MTEF@5@5@+=feaafiart1ev1aaatCvAUfKttLearuWrP9MDH5MBPbIqV92AaeXatLxBI9gBaebbnrfifHhDYfgasaacH8akY=wiFfYdH8Gipec8Eeeu0xXdbba9frFj0=OqFfea0dXdd9vqai=hGuQ8kuc9pgc9s8qqaq=dirpe0xb9q8qiLsFr0=vr0=vr0dc8meaabaqaciaacaGaaeqabaqabeGadaaakeaaiiGacqWFXoqydaqhaaWcbaGaemyta0eabaGaey4fIOcaaaaa@3091@ (Figure 2), αE∗
 MathType@MTEF@5@5@+=feaafiart1ev1aaatCvAUfKttLearuWrP9MDH5MBPbIqV92AaeXatLxBI9gBaebbnrfifHhDYfgasaacH8akY=wiFfYdH8Gipec8Eeeu0xXdbba9frFj0=OqFfea0dXdd9vqai=hGuQ8kuc9pgc9s8qqaq=dirpe0xb9q8qiLsFr0=vr0=vr0dc8meaabaqaciaacaGaaeqabaqabeGadaaakeaaiiGacqWFXoqydaqhaaWcbaGaemyraueabaGaey4fIOcaaaaa@3081@ does not occur exclusively in the range [0.8,1]. The maximizations of relevance and antiredundancy which happen in the maximization of the Euclidean distance are not always given equal weights either ('equal' as in the sense denoted by *α *= 0.5), since αE∗
 MathType@MTEF@5@5@+=feaafiart1ev1aaatCvAUfKttLearuWrP9MDH5MBPbIqV92AaeXatLxBI9gBaebbnrfifHhDYfgasaacH8akY=wiFfYdH8Gipec8Eeeu0xXdbba9frFj0=OqFfea0dXdd9vqai=hGuQ8kuc9pgc9s8qqaq=dirpe0xb9q8qiLsFr0=vr0=vr0dc8meaabaqaciaacaGaaeqabaqabeGadaaakeaaiiGacqWFXoqydaqhaaWcbaGaemyraueabaGaey4fIOcaaaaa@3081@ decreases as *K *increases and is not constantly located in the range [0.4,0.6] (Figure 1). Also, factors which have been oversimplified by the previous two assumptions (which state that Δ*d *is constant for all members of the predictor set) might contribute to the observation in Figure 1. Indeed, this aspect of the analysis provides us with the scope for future study on the differences between the two distances in the context of the DDP.

### Causes of the discrepancies between the plots for toy datasets and real-life datasets

The causes of the discrepancies between the plots for toy datasets and real-life datasets are based on the differences in dataset characteristics between toy datasets and real-life datasets.

• Small class sizes: compared to the class sizes in toy datasets (100 samples per class), some classes in the real-life datasets are comparatively small (e.g., 5 samples in the central nervous system tumor type in the NCI60 dataset).

• Varying class sizes: in toy datasets, the class size is kept fixed for all classes and all datasets (*m *= 100), whereas class size varies in real-life datasets. For example, the lung cancer class consists of 47 samples, whereas there are only 6 bladder tumor samples in the BRN dataset.

• Heterogeneity of some of the classes and the residual noise (variance within class) which remain even after discarding the fourth and the subsequent PCs: as mentioned previously, variance within class in real-life datasets, unlike the variance within class in toy datasets (which is fixed at 1), differs from class to class even within the same dataset.

Despite the aforementioned discrepancies and their probable causes, the general trends in the αE∗
 MathType@MTEF@5@5@+=feaafiart1ev1aaatCvAUfKttLearuWrP9MDH5MBPbIqV92AaeXatLxBI9gBaebbnrfifHhDYfgasaacH8akY=wiFfYdH8Gipec8Eeeu0xXdbba9frFj0=OqFfea0dXdd9vqai=hGuQ8kuc9pgc9s8qqaq=dirpe0xb9q8qiLsFr0=vr0=vr0dc8meaabaqaciaacaGaaeqabaqabeGadaaakeaaiiGacqWFXoqydaqhaaWcbaGaemyraueabaGaey4fIOcaaaaa@3081@ - *K*, αU∗
 MathType@MTEF@5@5@+=feaafiart1ev1aaatCvAUfKttLearuWrP9MDH5MBPbIqV92AaeXatLxBI9gBaebbnrfifHhDYfgasaacH8akY=wiFfYdH8Gipec8Eeeu0xXdbba9frFj0=OqFfea0dXdd9vqai=hGuQ8kuc9pgc9s8qqaq=dirpe0xb9q8qiLsFr0=vr0=vr0dc8meaabaqaciaacaGaaeqabaqabeGadaaakeaaiiGacqWFXoqydaqhaaWcbaGaemyvaufabaGaey4fIOcaaaaa@30A1@ - *K*, and αMP∗
 MathType@MTEF@5@5@+=feaafiart1ev1aaatCvAUfKttLearuWrP9MDH5MBPbIqV92AaeXatLxBI9gBaebbnrfifHhDYfgasaacH8akY=wiFfYdH8Gipec8Eeeu0xXdbba9frFj0=OqFfea0dXdd9vqai=hGuQ8kuc9pgc9s8qqaq=dirpe0xb9q8qiLsFr0=vr0=vr0dc8meaabaqaciaacaGaaeqabaqabeGadaaakeaaiiGacqWFXoqydaqhaaWcbaGaemyta0KaemiuaafabaGaey4fIOcaaaaa@31BA@ - *K *plots for real-life datasets in Figure 5 can be said to correspond to the general trends in the corresponding plots for toy datasets (Figures 1, 3, and 4). The overall picture provided by Figure 5 indicates that the effect of *K *on the values of the DDP which optimize d¯E,α
 MathType@MTEF@5@5@+=feaafiart1ev1aaatCvAUfKttLearuWrP9MDH5MBPbIqV92AaeXatLxBI9gBaebbnrfifHhDYfgasaacH8akY=wiFfYdH8Gipec8Eeeu0xXdbba9frFj0=OqFfea0dXdd9vqai=hGuQ8kuc9pgc9s8qqaq=dirpe0xb9q8qiLsFr0=vr0=vr0dc8meaabaqaciaacaGaaeqabaqabeGadaaakeaacuWGKbazgaqeamaaBaaaleaacqWGfbqrcqGGSaaliiGacqWFXoqyaeqaaaaa@31DA@, d¯MP,α
 MathType@MTEF@5@5@+=feaafiart1ev1aaatCvAUfKttLearuWrP9MDH5MBPbIqV92AaeXatLxBI9gBaebbnrfifHhDYfgasaacH8akY=wiFfYdH8Gipec8Eeeu0xXdbba9frFj0=OqFfea0dXdd9vqai=hGuQ8kuc9pgc9s8qqaq=dirpe0xb9q8qiLsFr0=vr0=vr0dc8meaabaqaciaacaGaaeqabaqabeGadaaakeaacuWGKbazgaqeamaaBaaaleaacqWGnbqtcqWGqbaucqGGSaaliiGacqWFXoqyaeqaaaaa@3313@, and U′Sα
 MathType@MTEF@5@5@+=feaafiart1ev1aaatCvAUfKttLearuWrP9MDH5MBPbIqV92AaeXatLxBI9gBaebbnrfifHhDYfgasaacH8akY=wiFfYdH8Gipec8Eeeu0xXdbba9frFj0=OqFfea0dXdd9vqai=hGuQ8kuc9pgc9s8qqaq=dirpe0xb9q8qiLsFr0=vr0=vr0dc8meaabaqaciaacaGaaeqabaqabeGadaaakeaacuWGvbqvgaqbamaaBaaaleaacqWGtbWudaWgaaadbaacciGae8xSdegabeaaaSqabaaaaa@3124@ in real-life datasets is the same as the effect in toy datasets.

## Conclusion

For each dataset from both collections of toy datasets and real-life microarray datasets, we have shown that there exists a value of the DDP which optimizes several characteristics representing the goodness of the predictor set. In turn, this optimal value of the DDP is influenced by the number of classes in the dataset.

We have also demonstrated, through selected examples from toy datasets and comparisons against both simplistic rank-based selection and state-of-the-art equal-priorities scoring methods, how the DDP concept works for datasets with different number of classes. A predictor set obtained at the optimal value of the DDP contains representative genes from *more *groups of marker genes than predictor sets found using any other values of the DDP. Thus the predictor set obtained using the optimal value of the DDP contains lower redundancy, and is capable of telling apart samples from more classes than predictor sets found using other, sub-optimal values of the DDP.

These findings have been achieved without turning to empirical experiments involving inductions of classifiers (which have previously proved the usefulness of the DDP for both artificial and real-life datasets), thus establishing the fundamental underpinnings for the DDP concept.

## Methods

### The DDP-based feature selection technique

For gene expression datasets, the terms gene and feature may be used interchangeably. From the total of *N *genes, the objective is to form the subset of genes, called the predictor set *S*, which gives the optimal classification accuracy.

The score of goodness for predictor set *S *is given as follows:

WA,S=(VS)α⋅(US)1−α
 MathType@MTEF@5@5@+=feaafiart1ev1aaatCvAUfKttLearuWrP9MDH5MBPbIqV92AaeXatLxBI9gBaebbnrfifHhDYfgasaacH8akY=wiFfYdH8Gipec8Eeeu0xXdbba9frFj0=OqFfea0dXdd9vqai=hGuQ8kuc9pgc9s8qqaq=dirpe0xb9q8qiLsFr0=vr0=vr0dc8meaabaqaciaacaGaaeqabaqabeGadaaakeaacqWGxbWvdaWgaaWcbaGaemyqaeKaeiilaWIaem4uamfabeaakiabg2da9maabmaabaGaemOvay1aaSbaaSqaaiabdofatbqabaaakiaawIcacaGLPaaadaahaaWcbeqaaGGaciab=f7aHbaakiabgwSixpaabmaabaGaemyvau1aaSbaaSqaaiabdofatbqabaaakiaawIcacaGLPaaadaahaaWcbeqaaiabigdaXiabgkHiTiab=f7aHbaaaaa@4248@

*V*_*S *_represents the relevance of *S*, while *U*_*S *_represents the antiredundancy of *S*. *V*_*S *_measures the average of the correlation of the members of *S *to the target class concept:

VS=1|S|∑i∈SF(i)
 MathType@MTEF@5@5@+=feaafiart1ev1aaatCvAUfKttLearuWrP9MDH5MBPbIqV92AaeXatLxBI9gBaebbnrfifHhDYfgasaacH8akY=wiFfYdH8Gipec8Eeeu0xXdbba9frFj0=OqFfea0dXdd9vqai=hGuQ8kuc9pgc9s8qqaq=dirpe0xb9q8qiLsFr0=vr0=vr0dc8meaabaqaciaacaGaaeqabaqabeGadaaakeaacqWGwbGvdaWgaaWcbaGaem4uamfabeaakiabg2da9maalaaabaGaeGymaedabaWaaqWaaeaacqWGtbWuaiaawEa7caGLiWoaaaWaaabuaeaacqWGgbGrdaqadaqaaiabdMgaPbGaayjkaiaawMcaaaWcbaGaemyAaKMaeyicI4Saem4uamfabeqdcqGHris5aaaa@3FC7@

The target class concept is represented by the target class vector **y**, which is defined as [*y*_1_, *y*_2_,..., *y*_|*T*|_], *y*_*j *_∈ [1, *K*] in a *K*-class dataset. *y*_*j *_is the class label of sample *j*. The training set, *T*, consists of all training samples of *K *classes. Based on **y**, the relevance of gene *i *is computed as follows:

F(i)=∑j∈T∑k=1KI(yj=k)(x¯i,k−x¯i,·)2∑j∈T∑k=1KI(yj=k)(xi,j−x¯i,k)2
 MathType@MTEF@5@5@+=feaafiart1ev1aaatCvAUfKttLearuWrP9MDH5MBPbIqV92AaeXatLxBI9gBaebbnrfifHhDYfgasaacH8akY=wiFfYdH8Gipec8Eeeu0xXdbba9frFj0=OqFfea0dXdd9vqai=hGuQ8kuc9pgc9s8qqaq=dirpe0xb9q8qiLsFr0=vr0=vr0dc8meaabaqaciaacaGaaeqabaqabeGadaaakeaacqWGgbGrdaqadaqaaiabdMgaPbGaayjkaiaawMcaaiabg2da9maalaaabaWaaabuaeaadaaeWbqaaiabdMeajnaabmaabaGaemyEaK3aaSbaaSqaaiabdQgaQbqabaGccqGH9aqpcqWGRbWAaiaawIcacaGLPaaadaqadaqaaiqbdIha4zaaraWaaSbaaSqaaiabdMgaPjabcYcaSiabdUgaRbqabaGccqGHsislcuWG4baEgaqeamaaBaaaleaacqWGPbqAcqGGSaalcqWIpM+zaeqaaaGccaGLOaGaayzkaaWaaWbaaSqabeaacqaIYaGmaaaabaGaem4AaSMaeyypa0JaeGymaedabaGaem4saSeaniabggHiLdaaleaacqWGQbGAcqGHiiIZcqWGubavaeqaniabggHiLdaakeaadaaeqbqaamaaqahabaGaemysaK0aaeWaaeaacqWG5bqEdaWgaaWcbaGaemOAaOgabeaakiabg2da9iabdUgaRbGaayjkaiaawMcaamaabmaabaGaemiEaG3aaSbaaSqaaiabdMgaPjabcYcaSiabdQgaQbqabaGccqGHsislcuWG4baEgaqeamaaBaaaleaacqWGPbqAcqGGSaalcqWGRbWAaeqaaaGccaGLOaGaayzkaaWaaWbaaSqabeaacqaIYaGmaaaabaGaem4AaSMaeyypa0JaeGymaedabaGaem4saSeaniabggHiLdaaleaacqWGQbGAcqGHiiIZcqWGubavaeqaniabggHiLdaaaaaa@7958@

where *I*(.) is an indicator function returning 1 if the condition inside the parentheses is true, otherwise it returns 0. x¯i,·
 MathType@MTEF@5@5@+=feaafiart1ev1aaatCvAUfKttLearuWrP9MDH5MBPbIqV92AaeXatLxBI9gBaebbnrfifHhDYfgasaacH8akY=wiFfYdH8Gipec8Eeeu0xXdbba9frFj0=OqFfea0dXdd9vqai=hGuQ8kuc9pgc9s8qqaq=dirpe0xb9q8qiLsFr0=vr0=vr0dc8meaabaqaciaacaGaaeqabaqabeGadaaakeaacuWG4baEgaqeamaaBaaaleaacqWGPbqAcqGGSaalcqWIpM+zaeqaaaaa@3314@ is the average of the expression of gene *i *across all training samples in *T*. x¯i,k
 MathType@MTEF@5@5@+=feaafiart1ev1aaatCvAUfKttLearuWrP9MDH5MBPbIqV92AaeXatLxBI9gBaebbnrfifHhDYfgasaacH8akY=wiFfYdH8Gipec8Eeeu0xXdbba9frFj0=OqFfea0dXdd9vqai=hGuQ8kuc9pgc9s8qqaq=dirpe0xb9q8qiLsFr0=vr0=vr0dc8meaabaqaciaacaGaaeqabaqabeGadaaakeaacuWG4baEgaqeamaaBaaaleaacqWGPbqAcqGGSaalcqWGRbWAaeqaaaaa@3203@ is the average of the expression of gene *i *across training samples belonging to class *k*. *x*_*i*,*j *_is the expression of gene *i *in sample *j*. *F*(*i*) is the BSS/WSS (between-groups sum of squares/within-groups sum of squares) ratio first used in [13] for multiclass molecular classification. It indicates the gene's ability in discriminating among samples belonging to *K *different classes.

Antiredundancy, introduced in [9], is a measure opposite to redundancy in quality:

US=1|S|2∑i,j∈S,i≠j1−|R(i,j)|
 MathType@MTEF@5@5@+=feaafiart1ev1aaatCvAUfKttLearuWrP9MDH5MBPbIqV92AaeXatLxBI9gBaebbnrfifHhDYfgasaacH8akY=wiFfYdH8Gipec8Eeeu0xXdbba9frFj0=OqFfea0dXdd9vqai=hGuQ8kuc9pgc9s8qqaq=dirpe0xb9q8qiLsFr0=vr0=vr0dc8meaabaqaciaacaGaaeqabaqabeGadaaakeaacqWGvbqvdaWgaaWcbaGaem4uamfabeaakiabg2da9maalaaabaGaeGymaedabaWaaqWaaeaacqWGtbWuaiaawEa7caGLiWoadaahaaWcbeqaaiabikdaYaaaaaGcdaaeqbqaaiabigdaXiabgkHiTmaaemaabaGaemOuai1aaeWaaeaacqWGPbqAcqGGSaalcqWGQbGAaiaawIcacaGLPaaaaiaawEa7caGLiWoaaSqaaiabdMgaPjabcYcaSiabdQgaQjabgIGiolabdofatjabcYcaSiabdMgaPjabgcMi5kabdQgaQbqab0GaeyyeIuoaaaa@4FDE@

The absolute value of the Pearson product moment correlation coefficient between genes *i *and *j*, |*R*(*i*,*j*)|, is used to measure the redundancy of gene *i *w.r.t. gene *j *(and vice-versa).

The power factor *α *∈ (0, 1] in equation (17) denotes the DDP between maximizing relevance and maximizing antiredundancy. We posit that different datasets will require different values of the DDP between maximizing relevance and maximizing antiredundancy in order to come up with the most efficacious predictor set. Therefore the optimal range of *α *(leading to the predictor set giving the best accuracy) is dataset-specific.

The linear incremental search [2,8] is conducted as follows: The first member of *S *is chosen by selecting the gene with the highest *F*(*i*) score. To find the second and the subsequent members of the predictor set, the remaining genes are screened one by one for the gene that gives the maximum *W*_*A*,*S*_. Since the combination of our predictor set scoring method and this search method does not specify an output as to the final size of the predictor set, *P*, the value of *P *will have to be predetermined by the user.

### Real-life datasets

Descriptions of eight real-life microarray datasets are shown in Table 3. The Brown (BRN) dataset [16] includes 15 broad cancer types. Following a previous study [17], the skin tissue samples due to small class size (3 samples) are excluded from analysis. The GCM dataset [6] contains 14 tumor classes. For the NCI60 dataset [18], only 8 tumor classes are analyzed; the 2 samples of the prostate class are excluded due to the small class size.

**Table 3:** Descriptions of real-life datasets. *N *is the number of features after preprocessing. *K *is the number of classes in the dataset.

Dataset	Type	*N*	*K*	Training set size:Test set size
BRN	cDNA	7452	14	174:83
GCM	Affymetrix	10820	14	144:54
NCI60	cDNA	7386	8	40:20
PDL	Affymetrix	12011	6	166:82
Lung	Affymetrix	1741	5	135:68
SRBC	cDNA	2308	4	55:28
MLL	Affymetrix	8681	3	48:24
AML/ALL	Affymetrix	3571	3	48:24

The PDL dataset [19] consists of 6 classes, each class representing a diagnostic group of childhood leukemia. The SRBC dataset [20] consists of 4 subtypes of small, round, blue cell tumors (SRBCTs). In the 5-class lung dataset [21], 4 classes are subtypes of lung cancer; the fifth class consists of normal samples. The MLL dataset [22] contains 3 subtypes of leukemia: ALL, MLL, and AML. The AML/ALL dataset [23] also contains 3 subtypes of leukemia: AML, B-cell, and T-cell ALL.

Except for the BRN and SRBC datasets (which are only available as preprocessed in their originating studies), datasets are preprocessed and normalized based on the recommended procedures [13] for Affymetrix and cDNA microarray data. Except for the GCM dataset, for which the ratio of training set size to test set size used in the originating study [6] is maintained to enable comparison with previous studies, for all datasets we employ the standard 2:1 split ratio.

A glossary of terms used in this manuscript is shown in Table 4

## Competing interests

The author(s) declare that they have no competing interests.

## Authors' contributions

CHO designed and implemented the DDP-based feature selection technique and the subsequent analyses on the technique, and drafted the manuscript. MC supervised and SWT co-supervised the study. Both MC and SWT provided inputs on the algorithm implementation and substantial edits on the manuscript. All authors read and approved the final manuscript.

**Table 4:** Glossary

**Term**	**Meaning**
αE∗ MathType@MTEF@5@5@+=feaafiart1ev1aaatCvAUfKttLearuWrP9MDH5MBPbIqV92AaeXatLxBI9gBaebbnrfifHhDYfgasaacH8akY=wiFfYdH8Gipec8Eeeu0xXdbba9frFj0=OqFfea0dXdd9vqai=hGuQ8kuc9pgc9s8qqaq=dirpe0xb9q8qiLsFr0=vr0=vr0dc8meaabaqaciaacaGaaeqabaqabeGadaaakeaaiiGacqWFXoqydaqhaaWcbaGaemyraueabaGaey4fIOcaaaaa@3081@	The value of the DDP leading to the best separation of classes in terms of the Euclidean distance
αM∗ MathType@MTEF@5@5@+=feaafiart1ev1aaatCvAUfKttLearuWrP9MDH5MBPbIqV92AaeXatLxBI9gBaebbnrfifHhDYfgasaacH8akY=wiFfYdH8Gipec8Eeeu0xXdbba9frFj0=OqFfea0dXdd9vqai=hGuQ8kuc9pgc9s8qqaq=dirpe0xb9q8qiLsFr0=vr0=vr0dc8meaabaqaciaacaGaaeqabaqabeGadaaakeaaiiGacqWFXoqydaqhaaWcbaGaemyta0eabaGaey4fIOcaaaaa@3091@	The value of the DDP leading to the best separation of classes in terms of the Manhattan distance in original feature space
αU∗ MathType@MTEF@5@5@+=feaafiart1ev1aaatCvAUfKttLearuWrP9MDH5MBPbIqV92AaeXatLxBI9gBaebbnrfifHhDYfgasaacH8akY=wiFfYdH8Gipec8Eeeu0xXdbba9frFj0=OqFfea0dXdd9vqai=hGuQ8kuc9pgc9s8qqaq=dirpe0xb9q8qiLsFr0=vr0=vr0dc8meaabaqaciaacaGaaeqabaqabeGadaaakeaaiiGacqWFXoqydaqhaaWcbaGaemyvaufabaGaey4fIOcaaaaa@30A1@	The value of the DDP giving the largest antiredundancy in PC space
αMP∗ MathType@MTEF@5@5@+=feaafiart1ev1aaatCvAUfKttLearuWrP9MDH5MBPbIqV92AaeXatLxBI9gBaebbnrfifHhDYfgasaacH8akY=wiFfYdH8Gipec8Eeeu0xXdbba9frFj0=OqFfea0dXdd9vqai=hGuQ8kuc9pgc9s8qqaq=dirpe0xb9q8qiLsFr0=vr0=vr0dc8meaabaqaciaacaGaaeqabaqabeGadaaakeaaiiGacqWFXoqydaqhaaWcbaGaemyta0KaemiuaafabaGaey4fIOcaaaaa@31BA@	The value of the DDP leading to the best separation of classes in terms of the Manhattan distance in PC space
*α*	A variable representing the DDP
Antiredundancy	A parameter opposite to redundancy in terms of quality and thus is to be maximized along with relevance
DDP	Degree of differential prioritization, which controls the balance between the two requirements in feature selection (maximizing relevance and maximizing antiredundancy)
Equal-priorities scoring methods	Filter-based feature selection techniques in which the predictor set scoring method places equal importance on relevance and redundancy as criteria in forming the predictor set
*G*	Number of genes in each group of marker genes, a parameter set during the generation of toy datasets
*K*	Number of classes in the dataset
*m*	Class size (number of samples per class), a parameter set during the generation of toy datasets
*N*	Number of genes in the dataset
OVA	One-vs.-all
*P*	Predictor set size, i.e., number of genes selected into the predictor set
PCA	Principal component analysis
PW	Pairwise
Rank-based selection or rank-based techniques	Filter-based feature selection techniques in which relevance is the sole criterion in forming the predictor set
Redundancy	The redundancy in a predictor set indicates the amount of similarity among the members of the predictor set
Relevance	The ability to distinguish among different classes
*S*_*α*_	The predictor set found using a DDP value of *α*
